# Cell wall mannan of *Candida krusei* mediates dendritic cell apoptosis and orchestrates Th17 polarization via TLR-2/MyD88-dependent pathway

**DOI:** 10.1038/s41598-018-35101-3

**Published:** 2018-11-20

**Authors:** Thu Ngoc Yen Nguyen, Panuwat Padungros, Panachai Wongsrisupphakul, Noppadol Sa-Ard-Iam, Rangsini Mahanonda, Oranart Matangkasombut, Min-Kyung Choo, Patcharee Ritprajak

**Affiliations:** 1 Graduate program in Oral Biology, Faculty of Dentistry, Chulalongkorn University, Bangkok, 10330 Thailand; 2 Organic Synthesis Research Unit, Department of Chemistry, Faculty of Science, Chulalongkorn University, Phayathai Road, Patumwan, Bangkok, 10330 Thailand; 3 Immunology Laboratory, Faculty of Dentistry, Chulalongkorn University, Bangkok, 10330 Thailand; 4 Department of Periodontology, Faculty of Dentistry, Chulalongkorn University, Bangkok, 10330 Thailand; 5 Laboratory of Biotechnology, Chulabhorn Research Institute, Bangkok, 10210 Thailand; 6 Research Unit on Oral Microbiology and Immunology and Department of Microbiology, Faculty of Dentistry, Chulalongkorn University, Bangkok, 10330 Thailand; 7 Cutaneous Biology Research Center, Massachusetts General Hospital and Harvard Medical School, Charlestown, MA 02129 USA; 8 Oral Biology Research Center, Faculty of Dentistry, Chulalongkorn University, Bangkok, Thailand

## Abstract

Dendritic cells (DCs) abundantly express diverse receptors to recognize mannans in the outer surface of *Candida* cell wall, and these interactions dictate the host immune responses that determine disease outcomes. *C. krusei* prevalence in candidiasis worldwide has increased since this pathogen has developed multidrug resistance. However, little is known how the immune system responds to *C. krusei*. Particularly, the molecular mechanisms of the interplay between *C. krusei* mannan and DCs remain to be elucidated. We investigated how *C. krusei* mannan affected DC responses in comparison to *C. albicans*, *C. tropicalis* and *C. glabrata* mannan. Our results showed that only *C. krusei* mannan induced massive cytokine responses in DCs, and led to apoptosis. Although *C. krusei* mannan-activated DCs underwent apoptosis, they were still capable of initiating Th17 response. *C. krusei* mannan-mediated DC apoptosis was obligated to the TLR2 and MyD88 pathway. These pathways also controlled Th1/Th17 switching possibly by virtue of the production of the polarizing cytokines IL-12 and IL-6 by the *C. krusei* mannan activated-DCs. Our study suggests that TLR2 and MyD88 pathway in DCs are dominant for *C. krusei* mannan recognition, which differs from the previous reports showing a crucial role of C-type lectin receptors in *Candida* mannan sensing.

## Introduction

*Candida* species are the most common cause of opportunistic fungal infections in immunocompromised individuals, leading to illnesses ranging from non-life-threatening mucocutaneous lesions to systemically invasive infections. Over recent decades, the incidence of candidiasis worldwide has shifted from *Candida albicans* to non-*albicans Candida* species (NACs) due to the evolution of resistance to anti-fungal medications^1,2^.

*Candida krusei* is an emerging nosocomial fungal pathogen primarily found in patients with hematologic malignancies undergoing bone marrow transplantation^3–6^. In addition, the frequency of *C. krusei* in candiduria and mucocutaneous candidiasis in diabetic patients has significantly risen recently^7–9^. The prevalence of *C. krusei* has increased since it became a multidrug-resistant pathogen because of its intrinsic fluconazole resistance and decreased susceptibility to flucytosine, amphotericin B and echinocandins^2,5,10–13^. Furthermore, this has made *C.krusei* infections difficult to treat and led to a high mortality rate^2,14^. Despite its increasing importance, little is known regarding the immune system response to *C. krusei*.

Carbohydrate constituents of *Candida* cell walls play a pivotal role in triggering host immune responses, which in turn either protect against the fungal infection or facilitate fungal immune evasion^15–17^. Mannans are mannose polymers located in the outermost part of *Candida* cell walls; therefore, they may be the first component to interact with the immune system. As cell wall mannans are complex structures, elaborate immune mechanisms have evolved^16,17^. While studies have shown that mannans can induce anti-fungal protective immunity^18–20^, other reports have revealed that mannans are a significant virulence factor associated with the severity and pathogenesis of *Candida* infections^21,22^. Furthermore, high levels of mannans can be detected in the blood of invasive candidiasis patients and it has been related to disease onset and progression^23,24^.

Dendritic cells (DCs) are potent antigen-presenting cells that reside in both lymphoid and non-lymphoid tissues and act as sentinels of the immune system. Interactions between invading pathogens and DCs via pathogen-associated molecular patterns (PAMPs) pattern-recognition receptors (PRRs) provide the foundation that triggers adaptive immune responses^16,25^. DCs abundantly express C-type lectin receptors (CLRs) and Toll-like receptors (TLRs), many of which can bind to *Candida* mannans. The activation of different types of mannan-specific receptors leads to differential DC activation that subsequently dictates distinct T cell responses^16,17,25^. Recognition of *Candida* mannans by CLRs and TLRs on DCs depends on mannan structure and mannosyl composition. In general, *Candida* N-linked mannans are recognized by dectin-2, mincle, mannose receptor (MR or CD206) and DC-SIGN (CD209), while O-linked mannans are recognized by TLR-4^17^. Furthermore, the α-mannans preferentially engage with dectin-2 and dectin-3^20,26^, while the β-mannans specifically ligate to galectin-3, which mediates TLR-2 activation^27,28^. The interactions of *Candida* mannans with several CLRs expressed on DCs induce Syk activation, which consequently mediates innate resistance to systemic fungal infection and orchestrates the Th17 response^19,29,30^. However, some mannose residues mediates signal transduction via the TLR/MyD88–dependent pathway, and participates in host defense against *C. albicans* infection^31–33^.

To date, the role of *C. krusei* mannan in DC immunity is not clear. Since mannan structures and mannosyl composition in the cell wall of *Candida* species are highly diverse, we compared the effects of cell wall mannans extracted from *C. albicans*, *C. troplicalis*, *C. glabrata* and *C. krusei* on DCs, and T cell responses.

## Results

### *C. krusei* mannan induced DC maturation and triggered massive productions of pro-inflammatory cytokines

**Figure 1:**
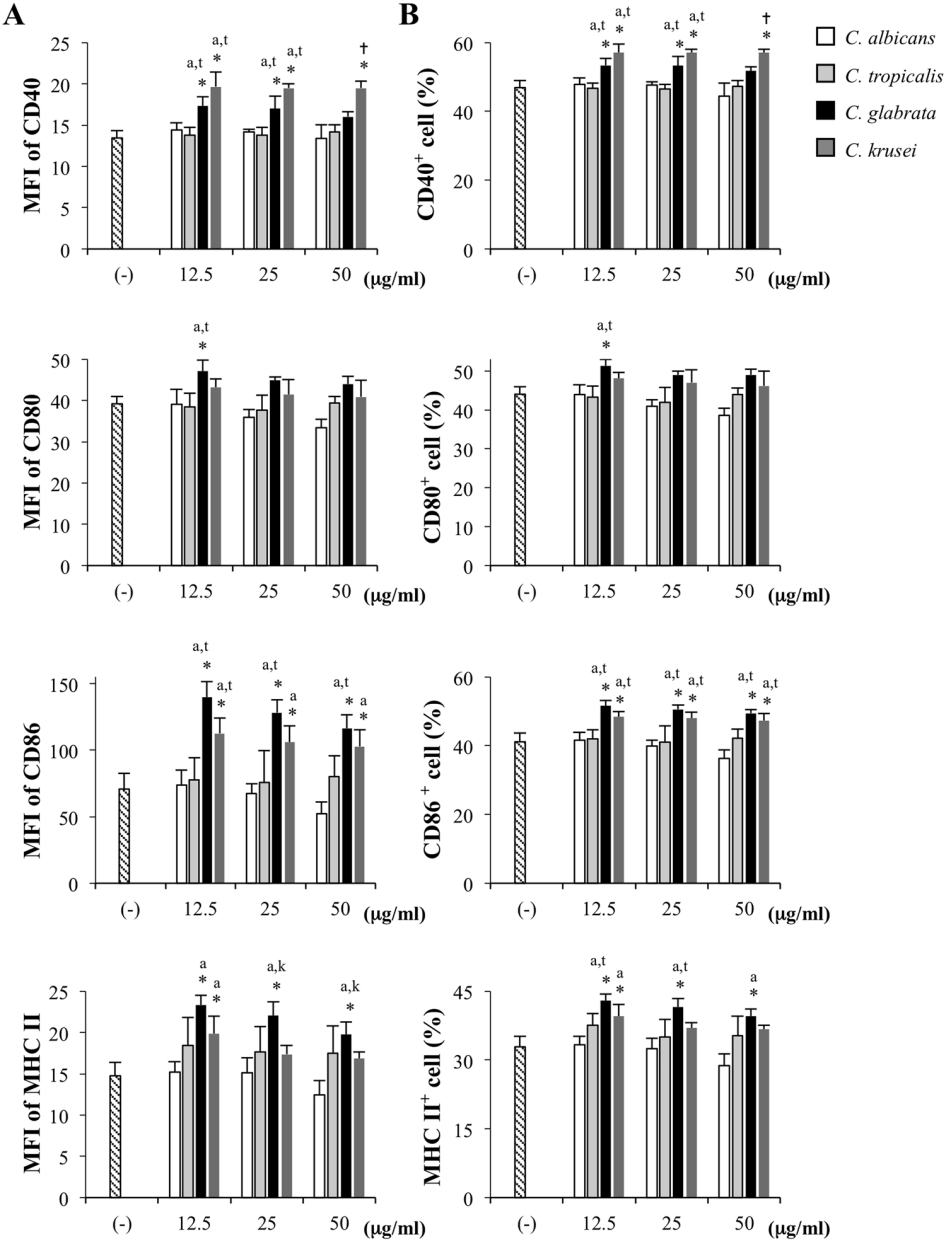
Differential phenotypic maturation of BMDCs in response to *Candida* mannan BMDCs were stimulated with *Candida* mannan at the indicated concentrations. Forty eight hours later, the expressions of CD11c, CD40, CD80, CD86 and MHC class II were assessed using flow cytometry as shown in Figs S1 and S2. Live BMDCs were defined based on side scatter (SSC) and forward scatter (FSC), and an electronic gate was placed on the CD11c^+^ cells. (**A**) the geometric mean fluorescence intensity (MFI) was evaluated using histogram analysis, and (**B**) the percentages of CD40^+^, CD80^+^, CD86^+^ and MHC class II^+^ cells within CD11c^+^ population were determined. *n* = 5; Data are representatives of two independent experiments. *p < 0.05 when compared to the unstimulated BMDCs, ^a^*p* < 0.05 when compared to *C. albicans*, ^t^*p* < 0.05 when compared to *C. tropicalis*, ^g^*p* < 0.05 when compared to *C. glabrata*, ^k^*p* < 0.05 when compared to *C. krusei*, ^†^*p* < 0.05 when compared to all groups; (−), unstimulated BMDCs; Ca, *C. albicans*; Ct, *C. tropicalis*; Cg, *C. glabrata*; Ck, *C. krusei*.

To evaluate whether cell wall mannans extracted from four distinct *Candida* species differentially affected the phenotypic maturation of DCs, BMDCs were stimulated with various concentrations of mannans and subsequently characterized by flow cytometric analyses of the maturation markers CD40, CD80, CD86 and MHC class II (Figs. 1, S1 and S2). The DC population was first identified by gating a DC marker, CD11c (Fig. S1A), and geometric MFI of the maturation markers was assessed using a histogram analysis (Figs. 1A and S1B). BMDCs stimulated with *C. albicans* and *C. tropicalis* mannans did not undergo maturation compared to the negative control, whereas those stimulated with *C. krusei* and *C. glabrata* mannans were potently activated. *C. krusei* mannan upregulated expression of CD40, CD86 and MHC class II on BMDCs, and induced the highest levels of CD40, especially at the highest mannan concentration. Although, *C. glabrata* mannan also induced CD80, CD86 and MHC Class II expression on BMDCs, expression differed slightly from that of BMDCs stimulated with *C. krusei* mannan. To determine the number of DCs that underwent maturation, dot plot analyses were performed to evaluate the percentage of CD11c^+^CD40^+^, CD11c^+^CD80^+^, CD11c^+^CD86^+^ and CD11c^+^MHC class II^+^ cells within the CD11c^+^ fraction (Figs 1B and S2). The percentages of each DC subpopulation were consistent with their geometric MFI levels (Figs. 1A and 1B).

**Figure 2:**
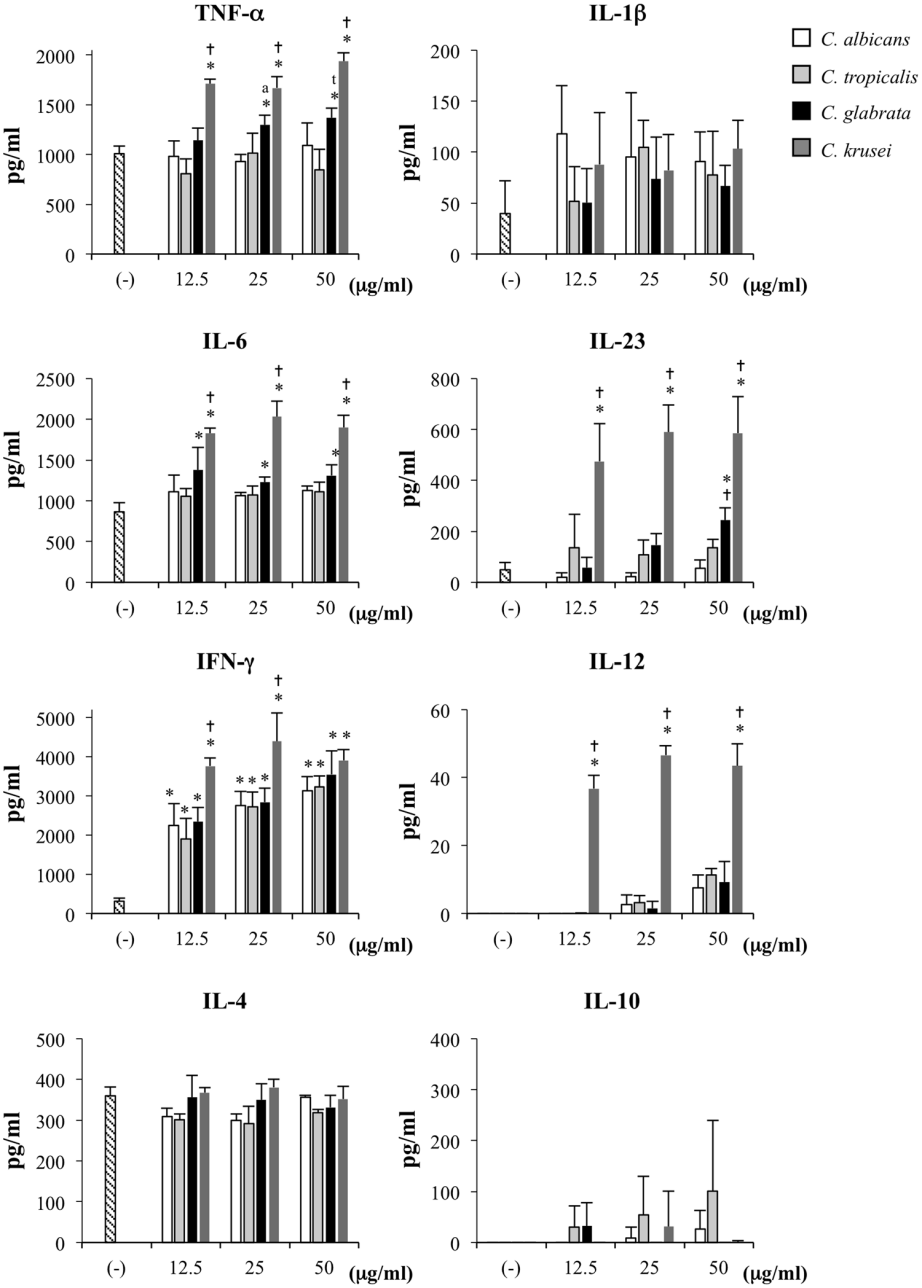
*C. krusei* mannan induced massive pro-inflammatory cytokine production. BMDCs were stimulated with *Candida* mannans at the indicated concentrations, and the cytokine productions in the culture supernatant were measured by ELISA. The levels of TNF-α, IL-6, IL-10 and IL-23 were detected at 24 h after stimulation, and the levels of IL-1β, IFN-γ, IL-12 and IL-4 were detected at 48 h after stimulation. *n* = 5; Data are representatives of two independent experiments. *p < 0.05 when compared to the unstimulated BMDCs, ^a^*p* < 0.05 when compared to *C. albicans*, ^t^*p* < 0.05 when compared to *C. tropicalis*, ^†^*p* < 0.05 when compared to all groups. (−), unstimulated BMDCs; Ca, *C. albicans*; Ct, *C. tropicalis*; Cg, *C. glabrata*; Ck, *C. krusei*.

Next, to determine the effects of mannan stimulation on DC cytokine production, we quantitated the levels of the pro-inflammatory cytokines TNF-α, IL-1β, IL-6, IL-23, IFN-γ, IL-12 and the anti-inflammatory cytokines IL-4 and IL-10 in response to *Candida* mannans (Fig. 2). *C. albicans* and *C. tropicalis* mannans induced significant levels of IFN-γ production in BMDCs compared to the negative control. However, in parallel to the effect on DC maturation, *C. albicans* and *C. tropicalis* mannans did not stimulate BMDCs to produce the other cytokines. *C. glabrata* mannan significantly upregulated IFN-γ, and slightly promoted IL-6 and IL-23 production; however, it failed to stimulate BMDCs to produce the other cytokines, regardless of its high DC-activation ability. Of note, *C. krusei* mannan at all concentrations augmented massive production of TNF-α, IL-6, IL-23, IFN-γ, and IL-12. None of the *Candida* mannans promoted production of the anti-inflammatory cytokines IL-4 and IL-10. Our findings imply that the cell wall mannans of these four distinct *Candida* species differentially impact DC maturation and function, and *C. krusei* mannan, in particular, elicits robust inflammatory DC responses.

### *C. kruse*i mannan reduced BMDC viability by the induction of cellular apoptosis

**Figure 3:**
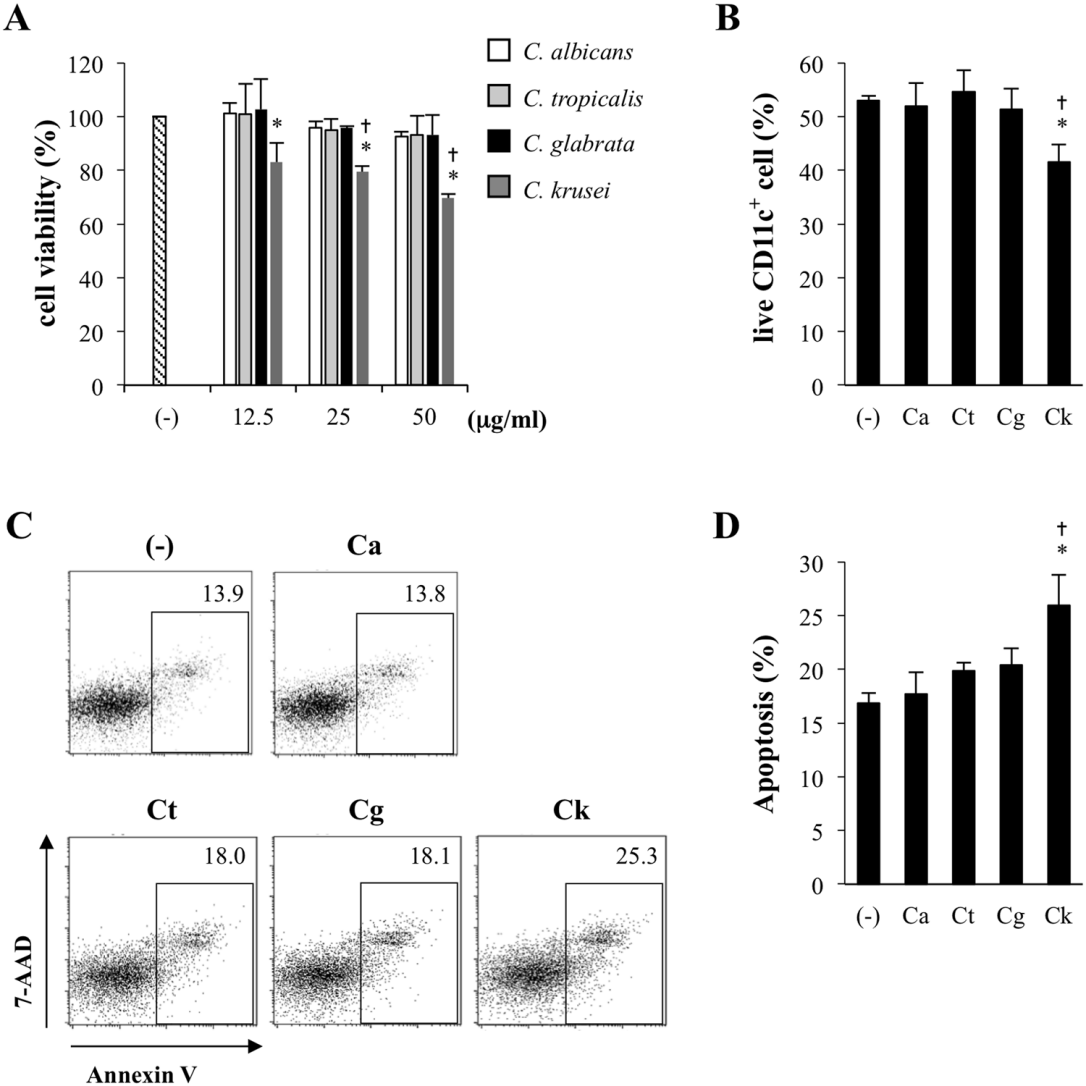
*C. krusei* mannan reduced DC viability by mediating DC apoptosis. (**A**) BMDCs were incubated with various concentrations of *Candida* mannans for 48 h, and the cell viability was determined by MTT assay. The percentage of cell viability was expressed as a percentage relative to the unstimulated BMDCs. (**B**) BMDCs were incubated with 25 μg/ml of *Candida* mannans for 72 h, and the percentage of live CD11c^+^ cells were determined (please see detail in Fig. S3). BMDCs were incubated with 25 μg/ml of *Candida* mannans for 48 h, then the cells were stained with CD11c, Annexin V and 7AAD. (**C**) CD11c^+^ cells were gated and the Annexin V^+^, and Annexin V^+^7AAD^+^ were identified as apoptotic fraction. The number in the dot plot indicated the percentage of apoptotic CD11c^+^ DCs. (**D**) the percentage of apoptotic CD11c^+^ cells was shown as the bar graph. *n* = 5; Data are representatives of two independent experiments. *p < 0.05 when compared to the unstimulated BMDCs, ^†^*p* < 0.05 when compared to all groups. (−), unstimulated BMDCs; Ca, *C. albicans*; Ct, *C. tropicalis*; Cg, *C. glabrata*; Ck, *C. krusei*.

Signal transduction via PRRs has been shown to lead to transcriptional expression of inflammatory cytokines and trigger cellular apoptosis^34,35^. On the basis of the vigorous pro-inflammatory cytokine production of BMDCs in response to *C. krusei* mannan stimulation, we hypothesized that *C. krusei* mannan may also induce cell death. To test this hypothesis, BMDCs were incubated with *Candida* mannans at various concentrations and cell viability was assessed by MTT assays. We found that *C. krusei* mannan at all concentrations markedly decreased BMDC viability, but the other *Candida* mannans did not (Fig. 3A). Changes in CD11c^+^ BMDCs were also observed using flow cytometric analysis. After CD11c^+^ cells were gated, live cells were identified based on forward scatter (FSC) and side scatter (SSC) signals (Fig. S3A). The percentage of live cells in the CD11c^+^ population was notably reduced when BMDCs were incubated with *C. krusei* mannan, whereas they were unaffected by mannans from the other three *Candida* species (Figs. 3B and S3B). To determine whether the reduction in live cells was due to DC apoptosis, unstimulated and *Candida*-mannan-stimulated BMDCs were stained with CD11c, Annexin V and 7AAD. CD11c^+^ cells that were single positive for Annexin V or double positive for Annexin V and 7AAD were identified as apoptotic DCs (Fig. 3C). Consistently, only *C. krusei* mannan significantly induced apoptosis in DCs (Fig. 3D). Our data imply that *C. krusei* mannan affects DC viability through a process of activation-induced cellular apoptosis.

### *C. krusei* mannan reduced BMDC viability by the induction mannan mediated DC apoptosis via MyD88-dependent pathway

**Figure 4:**
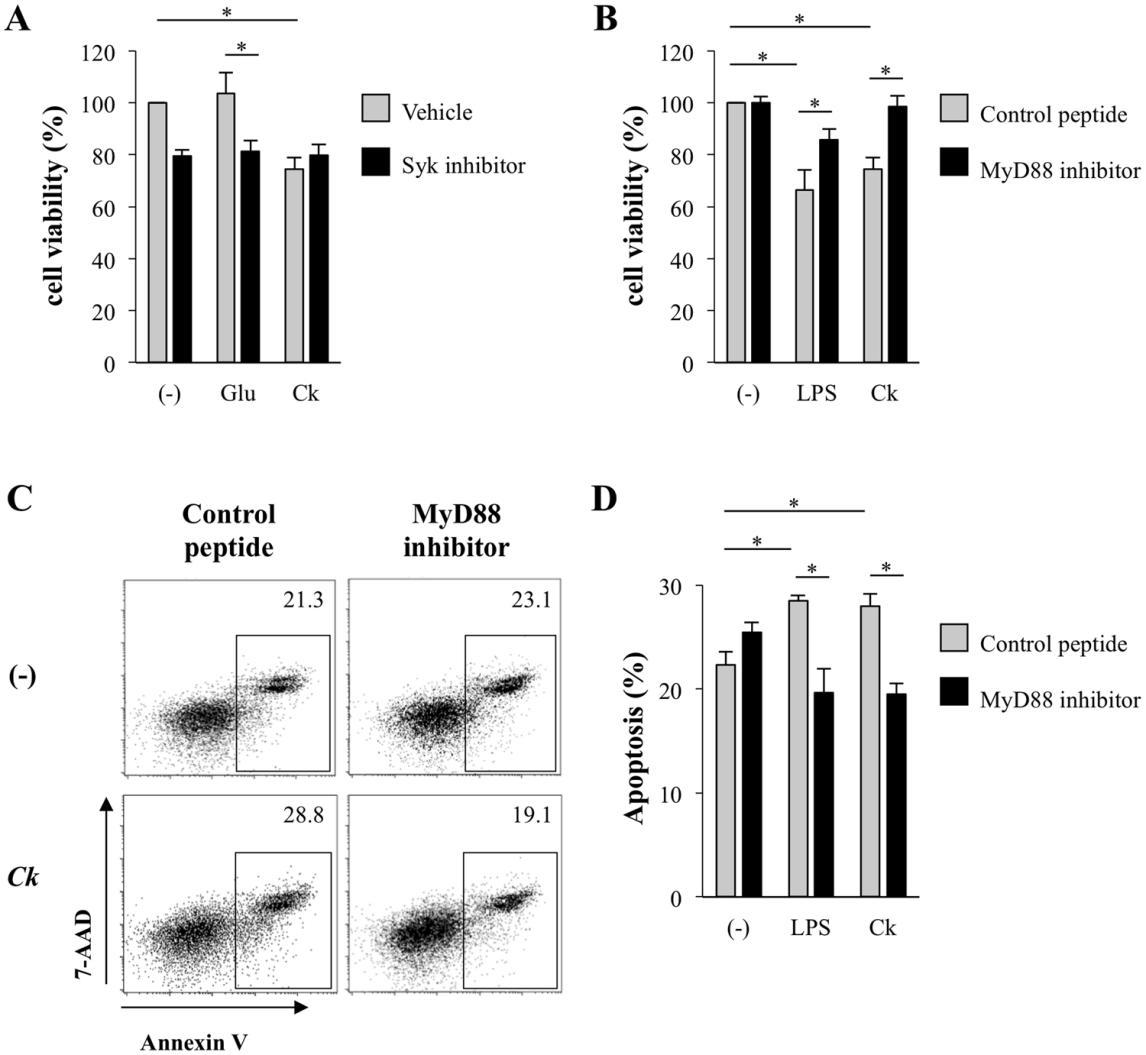
DC apoptosis induced by *C. krusei* mannan was dependent on MyD88 signaling pathway. (**A**) BMDCs were pre-treated with vehicle control or Syk inhibitor, and the cells were incubated with 25 μg/ml of β-glucan or 25 μg/ml of *C. krusei* mannan for 48 h. (**B**) BMDCs were pre-treated with control peptide or MyD88 inhibitor, and the cells were incubated with 0.5 μg/ml LPS or 25 μg/ml of *C. krusei* mannans for 48 h. The BMDC viability was determined by MTT assay. The percentage of cell viability was expressed as a percentage relative to the unstimulated BMDCs. (**C**) and (**D**) BMDCs were pre-treated with control peptide or MyD88 inhibitor, and the cells were incubated with 0.5 μg/ml of LPS or 25 μg/ml of *C. krusei* mannan for 48 h. Then, the cells were stained with CD11c, Annexin V and 7AAD. CD11c^+^ cells were gated and the Annexin V^+^, and Annexin V^+^7AAD^+^ were identified as apoptotic fraction. The number in the dot plot indicated the percentage of apoptotic DCs. (**D**) The percentage of apoptotic CD11c^+^ cells was shown as the bar graph. *n* = 3; Data are representatives of two independent experiments. *p < 0.05. (−), unstimulated BMDCs; Ca, *C. albicans*; Ct, *C. tropicalis*; Cg, *C. glabrata*; Ck, *C. krusei*.

C-type lectin receptors (CLRs) such as dectin-2, dectin-3 and mincle are the main receptors in recognizing *Candida* mannans; however, some mannan structures do engage with TLRs^16,17^. As previous studies have shown that the mannan structures of *Candida* species are diverse^36,37^, we questioned which pathway is responsible for *C. krusei* mannan-mediated DC apoptosis. To elucidate the signal transduction underlying this phenomenon, downstream signaling of the CLR-coupled Syk and TLR-MyD88 pathways was individually investigated using pathway-specific inhibitors. BMDCs were pre-treated with Syk or MyD88 inhibitors, and subsequently stimulated with *C. krusei* mannan. Cell viability was determined using MTT assay. *C. albicans* β-glucan was used as a positive control since it transduces signals via the dectin-1-Syk pathway^38^, and we found that it did not affect BMDC cell viability (Fig. 4A). Inhibition of Syk in unstimulated and β-glucan-stimulated BMDCs significantly decreased cell viability, but did not abrogate *C. krusei* mannan-induced cell death (Fig. 4A). In a parallel experiment to examine the role of MyD88, LPS was used as the positive control for MyD88 activation. LPS stimulation resulted in a marked reduction in BMDC viability, similarly to *C. krusei* mannan stimulation, and inhibition of MyD88 rescued the viability of BMDCs stimulated with *C. krusei* mannan as well as that of those stimulated with LPS (Fig. 4B). In addition, the apoptosis staining assay demonstrated that MyD88 inhibition, to a great extent, interfered with both LPS- and *C. krusei* mannan-mediated apoptosis of the CD11c^+^ population (Figs. 4C and 4D). These results imply an intriguing role of the MyD88-dependent signaling pathway in DC apoptosis in response to *C. krusei* mannan stimulation.

### DC apoptosis-mediated by C. krusei mannan was associated with TLR2 activation

**Figure 5:**
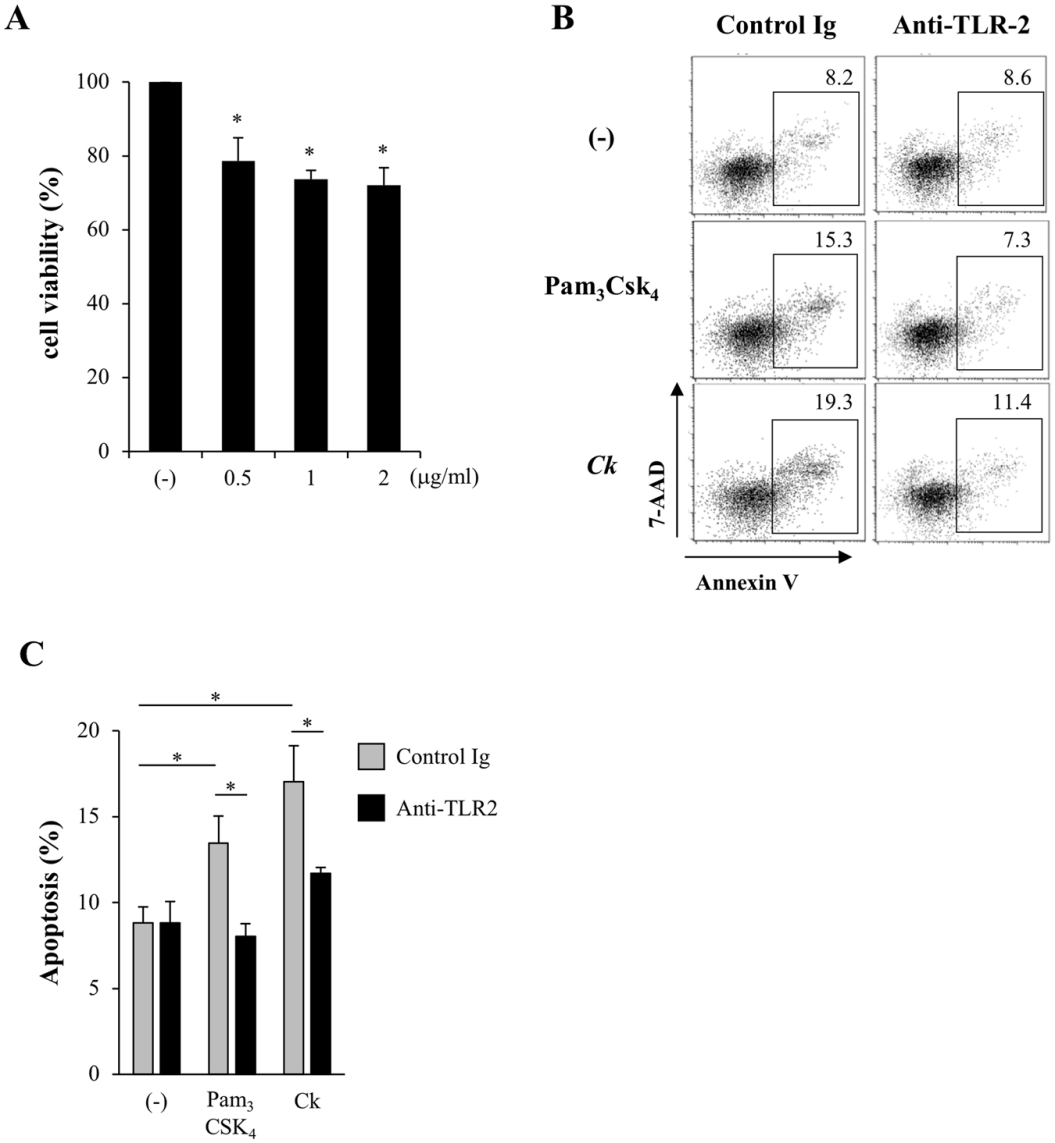
*C. krusei* mannan mediated DC apoptosis via the activation of TLR2. (**A**) BMDCs were incubated with various concentration of Pam_3_CSK_4_ as indicated for 48 h, and the cell viability was determined by MTT assay. The percentage of cell viability was expressed as a percentage relative to the unstimulated BMDCs. n = 4; Data are representatives of two independent experiments. *p < 0.05 when compared to the negative control. (**B**) and (**C**) BMDCs were pre-treated with control rat IgG or anti-mouse TLR2 mAbs, and the cells were incubated with 0.5 μg/ml of Pam_3_CSK_4_ or 25 μg/ml of *C. krusei* mannan for 48 h. Then, the cells were stained with CD11c, Annexin V and 7AAD. CD11c^+^ cells were gated and the Annexin V^+^, and Annexin V^+^7AAD^+^ were identified as apoptotic fraction as shown in (**B**). The number in the dot plot indicated the percentage of apoptotic CD11c^+^ DCs. (**C**) The percentage of apoptotic CD11c^+^ cells was shown as the bar graph. *n* = 3; Data are representatives of three independent experiments.*p < 0.05. (−), unstimulated BMDCs; Ca, *C. albicans*; Ct, *C. tropicalis*; Cg, *C. glabrata*; Ck, *C. krusei*.

Previous studies have shown that the mannosyl residuals of *C. albicans* cell wall can be recognized by TLR2^39^, and this recognition mediated macrophage apoptosis^40^. We therefore investigated the functional relevance of TLR2 in DC apoptosis triggered by *C. krusei* mannan stimulation. To confirm that the activation of TLR2 also induce the cell death, BMDCs were incubated with a specific TLR2 ligand, Pam_3_CSK_4_, and the cell viability was determined using MTT assay. As expected, BMDCs stimulated with Pam_3_CSK_4_ reduced the BMDC viability (Fig. 5A). Next, *C. krusei* mannan-mediated DC apoptosis via TLR2 ligation was determined by using the blocking antibodies. As a positive control, Pam_3_CSK_4_, significantly enhanced DC apoptosis, and this Pam_3_CSK_4_–induced cellular apoptosis was impeded by pre-treatment with the anti-TLR2 blocking antibody. Notably, blockade with anti-TLR2 mAbs merely diminished the effect of *C. krusei* mannan on DC apoptosis (Figs. 5B and 5C). These findings were concordant with the results of MyD88 inhibition (Fig. 4D).

We also observed the effect of *C. krusei* cell wall on DC apoptosis (Fig. S4). *C. krusei* yeast cells were inactivated by heat so that the cells could not secrete any molecules that might have influenced the DC response. In addition, we expected that mannans would be the first component to interact with DCs because they are located in the outermost layer of *Candida* cell walls^16,17^. Consistent with the apoptosis induction by *C. krusei* mannan (Fig. 5C), BMDCs stimulated by heat-inactivated *C. krusei* yeast cells also displayed significantly increased apoptosis, and blockade of TLR2 abrogated apoptosis induction (Fig. S4).

Taken together, our data indicate that TLR2 plays an important role in the mechanism of apoptosis induction by the cell wall mannan of *C. krusei*.

### C. krusei mannan-stimulated DCs orchestrated antigen-specific Th17 response

**Figure 6:**
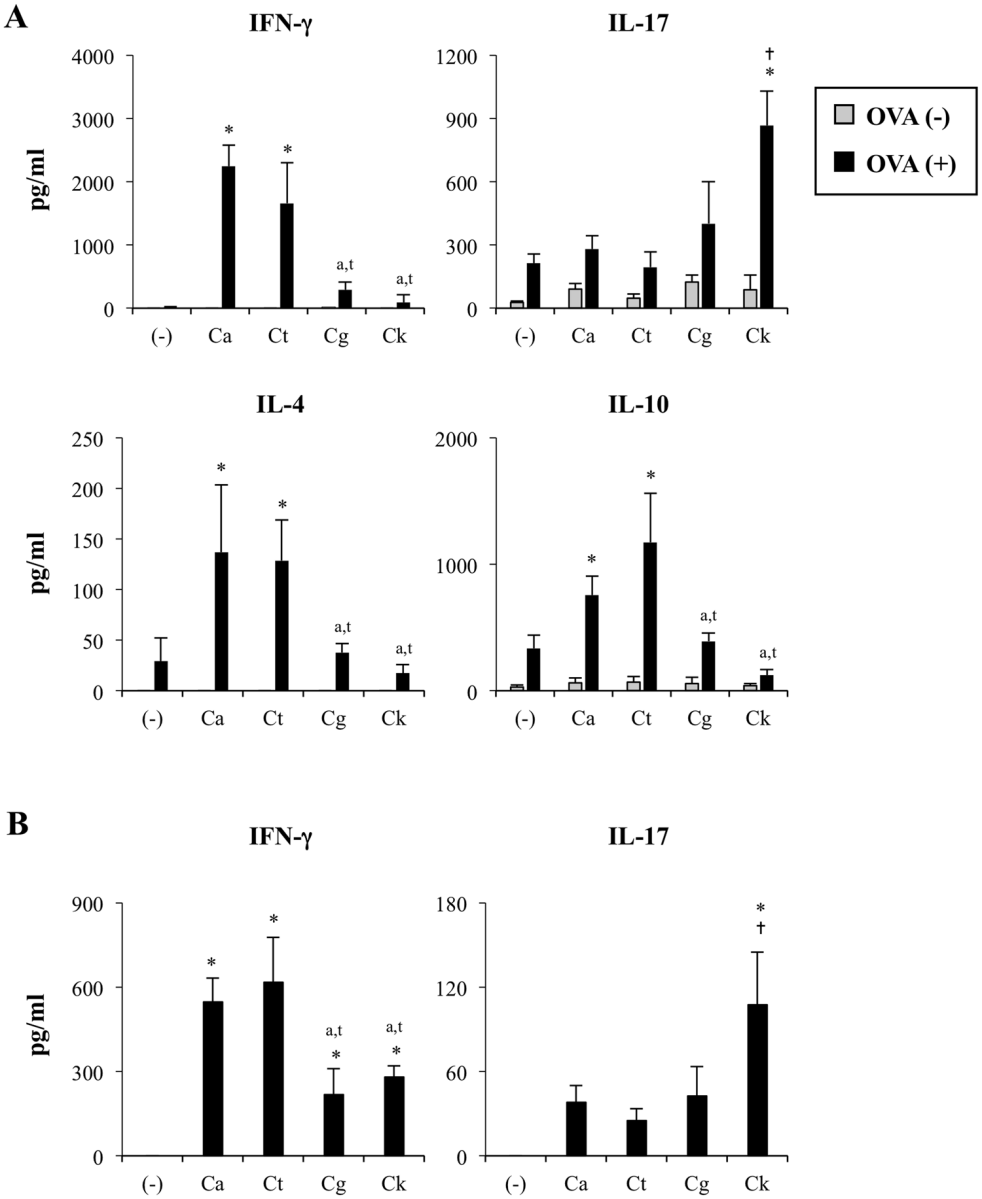
*C. krusei* mannan governed Th17 response in an antigen-specific manner. (**A**) Mice were subcutaneously immunized with the mixture of OVA and *Candida* mannan at day 0 and day 7. At day 14, the RLN cells were re-simulated with (+) or without (−) OVA, and the levels of IFN-γ, IL-17, IL-4 and IL-10 in the culture supernatant were quantitated by ELISA. *n* = 5; Data are representatives of two independent experiments. *p < 0.05 when compared to the negative control, ^a^*p* < 0.05 when compared to OVA-*C. albicans*, ^t^*p* < 0.05 when compared to OVA-*C. tropicalis*, ^†^*p* < 0.05 when compared to all groups. (−), Mice were immunized with OVA alone as a negative control; Ca, OVA-*C. albicans*; Ct, OVA-*C. tropicalis*; Cg, OVA-*C. glabrata*; Ck, OVA-*C. krusei*. (**B**) BMDCs were stimulated with 25 μg/ml of *Candida* mannan, and the cells were stimulated with 25 μg/ml of *C. albicans* or *C. krusei* mannan. Subsequently unstimulated and mannan-stimulated BMDCs were pulsed with OVA, and the BMDCs were then co-cultured with OT-II T cells for 48 h. The level of IFN-γ and IL-17 in the culture supernatant was determined by ELISA. *n* = 3; Data are representatives of two independent experiments. *p < 0.05 when compared to the negative control, ^a^*p* < 0.05 when compared to *C. albicans*, ^t^*p* < 0.05 when compared to *C. tropicalis*, ^†^*p* < 0.05 when compared to all groups; (−), T cells incubated with OVA were used as a negative control; Ca, *C. albicans*; Ct, *C. tropicalis*; Cg, *C. glabrata*; Ck, *C. krusei*.

Since *C. krusei* mannan induced massive cytokine production and impaired DC viability, we next investigated antigen-specific T cell responses to stimulated DCs. As a large number of DCs reside in the skin^41^, mice were immunized with a mixture of ovalbumin and various *Candida* mannans (OVA-*Candida* mannans) via a subcutaneous route so that DCs could be exposed directly to the stimuli. Seven days after the final immunization, the immune cell populations (T cells, B cells, memory T cells, and Th subsets) in regional lymph nodes (RLNs) were examined by flow cytometric analysis (Figs S5 and S6). Total numbers of RLN cells were significantly increased when mice were immunized with OVA-*C. albicans* mannan or OVA-*C. krusei* mannan. However, none of the immunized mice exhibited any alteration in the proportions of immune cell populations (Fig. S6). Several lines of evidence have revealed that *Candida* cell wall- and mannan-stimulated DCs can dictate the fate of CD4 T cell polarization^20,25^. Thus, we further examined T cell responses when stimulated *ex vivo*. The antigen-primed LN T cells were re-stimulated with OVA, and the levels of Th cytokines, IFN-γ, IL17, IL-4 and IL-10, were determined (Fig. 6A). Upon *ex vivo* antigen re-exposure, OVA-*C. albicans* mannan- and OVA-*C. tropicalis* mannan-immunized LN cells markedly upregulated IFN-γ, IL-4 and IL-10 secretion compared to the OVA-*C. glabrata* mannan, OVA-*C. krusei* mannan, and negative control groups. On the other hand, immunization with OVA-*C. glabrata* mannan did not activate cytokine production. Augmented IL-17 production was observed particularly in OVA-*C. krusei* mannan-immunized LN cells.

*In vivo* mannan immunization may not only activate DCs but also possibly affect the response of other immune cells. Therefore, to investigate whether the distinct T cell responses were due to the direct impact of *Candida* mannan-stimulated DCs on CD4 T cells, we performed an *in vitro* co-culture assay. BMDCs were primed with *Candida* mannans and subsequently pulsed with OVA. These BMDCs were then co-cultured with T cells isolated from OT-II TCR transgenic mice. Secretion of IFN-γ, IL-17, IL-4 and IL-10 in the culture supernatant, representing T helper cell responses, was analyzed. Concurring with our results from the *ex vivo* re-stimulation assays, *C. abicans* and *C. tropicalis* mannan-stimulated DCs profoundly augmented IFN-γ production from OT-II T cells, while *C. krusei* mannan-stimulated DCs preferentially induced IL-17 production (Fig. 6B). IL-4 and IL-10 were not detectable in this co-culture system. Collectively, our results reveal that DCs activated with *C. krusei* mannan, despite undergoing apoptosis, were capable of orchestrating the Th17 response in an antigen-specific manner.

### Th17 induction by *C. krusei* mannan-stimulated DCs was via the MyD88 signaling pathway

**Figure 7:**
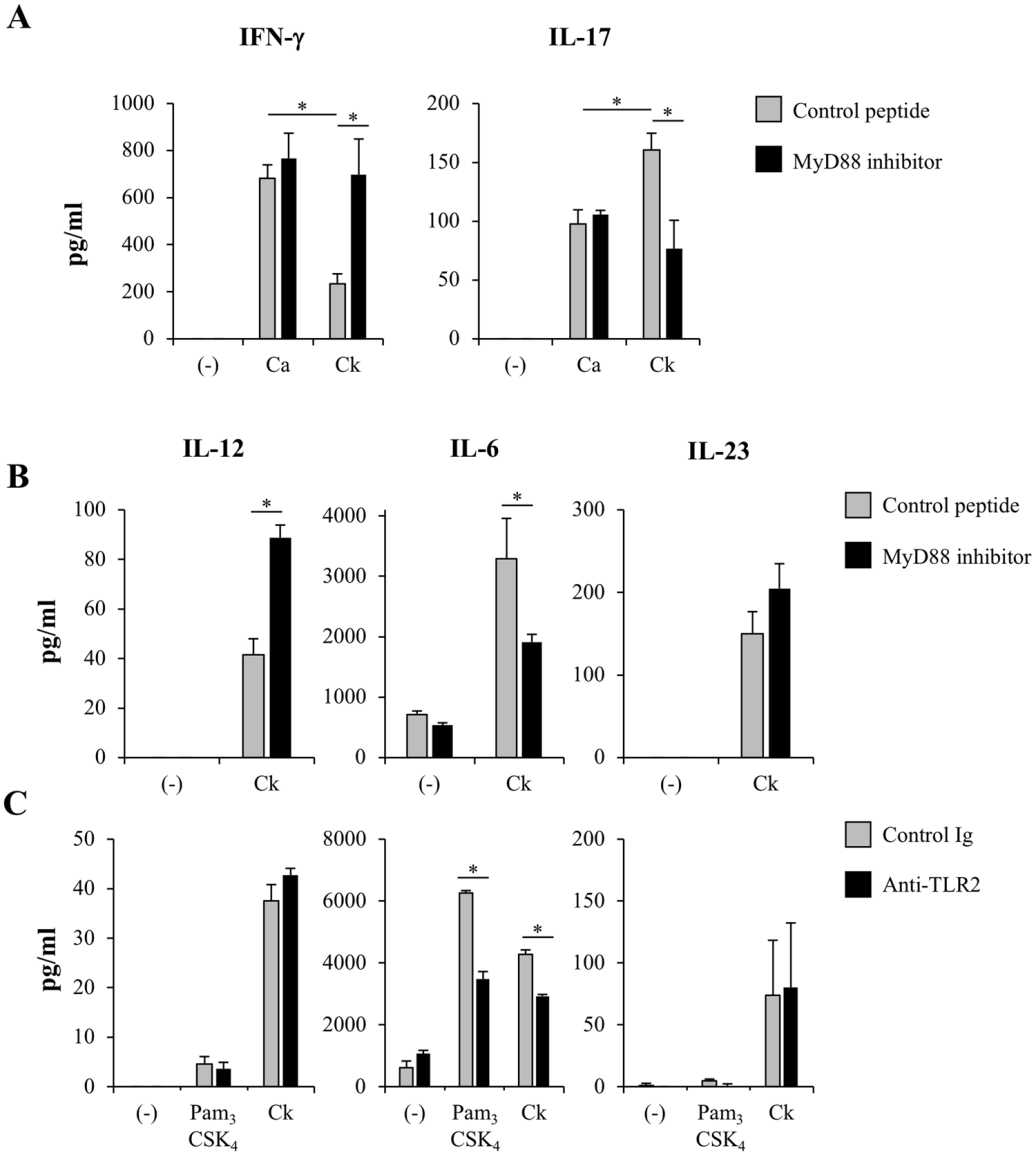
*C. krusei* mannan-stimulated DCs skewed Th1/Th17 polarization via MyD88 and TLR2 pathway. (**A**) BMDCs were pre-treated with control peptide or MyD88 inhibitor, and the cells were stimulated with 25 μg/ml of *C. albicans* or *C. krusei* mannan. Subsequently, unstimulated and mannan-stimulated BMDCs were pulsed with OVA, and the BMDCs were co-cultured with OT-II T cells for 48 h. The levels of IFN-γ and IL-17 in the culture supernatant were determined by ELISA. (−), T cell incubated with OVA as a negative control. n = 3; Data are representatives of two independent experiments. *p < 0.05. Ca, *C. albicans*; Ck, *C. krusei*. (**B**–**D**) BMDCs were pre-treated with (**B**) control peptide or MyD88 inhibitor (**C**) control IgG or anti-mouse TLR2 mAbs. Then, the cells were stimulated with 25 μg/ml of *C. krusei* mannan, and the levels of IL-12, IL-6 and IL-23 in the culture supernatant were determined by ELISA. *n* = 3; Data are representatives of three independent experiments. *p < 0.05. (−), unstimulated BMDCs; Ck, *C. krusei*.

Having demonstrated the involvement of the MyD88-dependent pathway in DC apoptotic response to *C. krusei* mannan (Fig. 4), we investigated whether MyD88 was also required for Th17 induction by *C. krusei* mannan-stimulated DCs. BMDCs were pre-treated with a MyD88 inhibitor and then primed with *C. albicans* mannan or *C. krusei* mannan. Subsequently, these BMDCs were pulsed with OVA and co-cultured with OT-II T cells. Inhibition of MyD88 in DCs stimulated with mannan from *C. krusei* converted the production of IL-17 to IFN-γ in the activated OT-II T cells, but this did not occur in DCs stimulated with mannan from *C. albicans* (Fig. 7A). TLR-2 blockade in *C. krusei* mannan-stimulated DCs also resulted in reduced IL-17 production by OT-II T cells, but did not affect IFN-γ production (Fig. S7A). Consistently, MyD88 inhibition in BMDCs stimulated with *C. krusei* mannan significantly reduced IL-6, the key cytokine in Th17 induction^42,43^, while it substantially enhanced the production of IL-12, which is required for Th1 induction^42^ (Fig. 7B).

We further verified the roles of TLR2 in the cytokine production of *C. krusei* mannan-stimulated DCs by using blocking antibodies. TLR2 blockade significantly suppressed production of IL-6, but not that of IL-12 and IL-23, in BMDCs stimulated with Pam_3_CSK_4_ and with *C. krusei* mannan (Fig. 7C). IL-1β is also play a role in Th17 polarization^44^, and TLR-2 stimulation has participated in the production of IL-1β^45^. However, we found that TLR-2 blockade did not alter IL-1β production in BMDCs stimulated with *C. krusei* mannan (Fig. S7B). This result may be due to the inability of *C. krusei* mannan to induce IL-1β production in BMDCs (Fig. 2).

To confirm the differences in cell wall mannan structure between *Candida* species, the mannan composition were analyzed using nuclear magnetic resonance spectroscopy (NMR). NMR characterizations of all *Candida* mannans in our study were consistent with previous reports^36,37,46–49^ (Figs S8–S11). Additionally, structural analysis of *C. krusei* mannan was further investigated (Fig. S11). ^13^C NMR spectrum contained signals at 103.31, 101.76, and 99.28 ppm corresponded to anomeric carbons (C1) of terminal non-reducing, 2-substituted, and 2,6-disubstituted mannose, respectively^48^ (Fig. S11A). Acidic hydrolysis of *C. krusei* mannan was then carried out to support the structural analysis. Acylation, acetolysis and O-deacetylation of *C. krusei* mannan, followed by gel filtration on Bio-Gel P-2 gave manno-oligosaccharide. ^1^H NMR spectrum of the manno-oligosaccharide showed signals at 5.36, 5.27, and 5.01 ppm for anomeric proton (H1) of non-reducing end, α-1,2-linked, and reducing end, respectively (Fig. S11B). This result indicated that *C. krusei* mannan possessed long chain of α−1,2-linked backbone, which resembled the structure reported previously^48,50^.

^1^H NMR spectra were supported the structural analysis. Signals between 5.60–4.90 ppm represented α-linked mannose residues (Fig. 12)^46^. Distinctive down filed signals of 3 mannans derived from *C. albicans* (5.48 ppm), *C. tropicalis* (5.48 and 5.46 ppm), and *C. glabrata* (5.57 and 5.58 ppm) indicated phosphodiesterified 1,2-linked mannose residues^51^. On the other hand, the down filed signal was not observed from ^1^H NMR of *C. krusei* mannan. This was consistent with the absence of phosphodiester linkage in mannan structure. ^1^H NMR signals of mannans with α-1,2-linkages were also detected, generally in down filed regions, such as *C. albicans* (5.32 ppm), *C. tropicalis* (5.20 ppm), *C. glabrata* (5.30 ppm), and *C. krusei* (5.21, 5.16, 5.14 ppm)^36^. Molecular weights of *Candida* mannans were also determined by using gel permeation chromatography (GPC) as follows; *C. albicans* mannan (925 kDa), *C. tropicalis* mannan (387 kDa), *C. glabrata* mannan (158 kDa), and *C. krusei* mannan (169 kDa) (Figs. S13–S16). The molecular weights were consistent with the proposed *Candida* mannan structures (Fig. S17).

**Figure 8:**
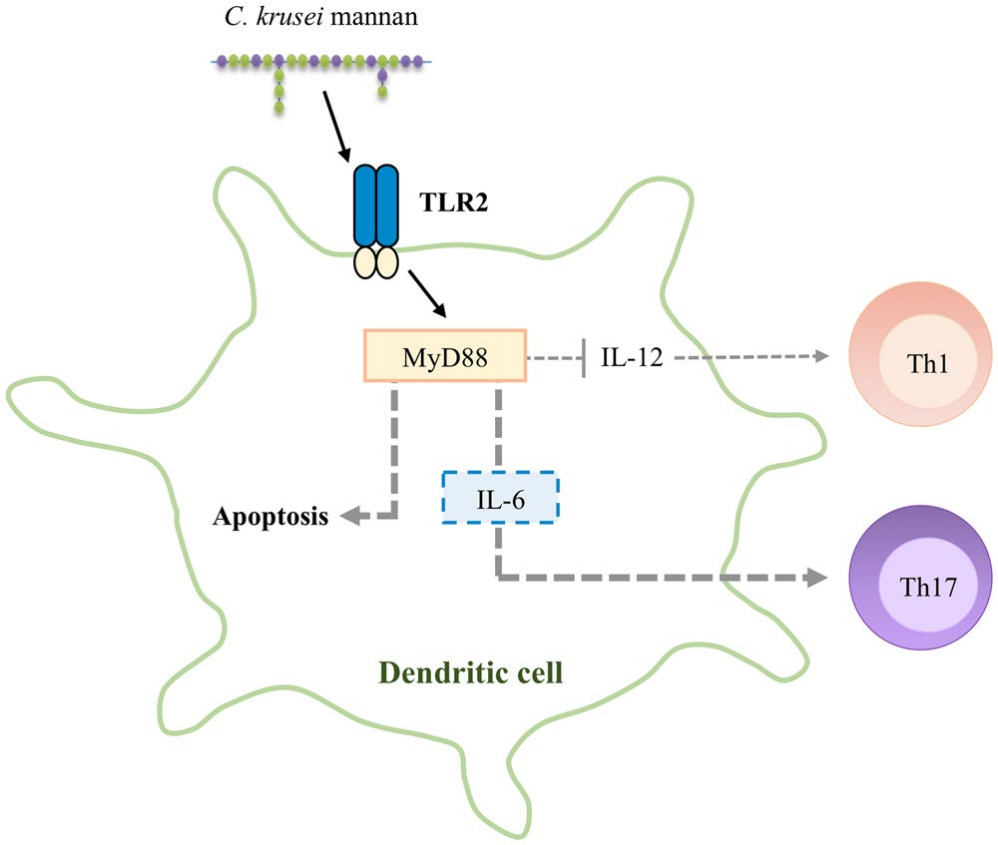
A schematic model of the signal transduction in DCs in response to *C. krusei* mannan *C. krusei* mannan activates TLR2/MyD88 signal in DCs and triggers apoptotic death. In parallel, the signal transduction through TLR2/MyD88 interferes with the production of IL-12 but promotes the production of IL-6, which consequently skews Th1/Th17 differentiation.

In summary, our findings indicate that DCs recognize *C. krusei* mannan via a TLR2/MyD88 dependent pathway, and this interaction results in apoptosis. Likewise, signal transduction through TLR2/MyD88 also regulates IL-12 and IL-6 production in DCs, which results in a shift toward Th1 and Th17 immunity, respectively (Fig. 8).

## Discussion

In this study, we provide evidence that DCs respond differently to cell wall mannans from different *Candida* species. We investigated the molecular mechanisms underlying the induction of DC and T-cell immunity in response to *C. krusei* cell wall mannan. In contrast to previous reports on mannans from other species, our results show that *C.krusei* mannan activates DCs and consequently guides Th cell responses through the TLR2/MyD88 pathway.

We examined the responses of DCs to the cell wall mannans of four *Candida* species that are commonly found as opportunistic pathogens in immunocompromised patients: *C. albicans*, *C. tropicalis*, *C. glabrata* and *C. krusei*. Several *Candida* species are dimorphic fungi that can switch between the yeast and filamentous forms in response to environmental conditions such as high temperature, pH, and nutrition, whereas other *Candida* species exist in only one form^52,53^. *C. albicans* and *C. tropicalis* have three forms with distinct morphology; yeast cell, pseudohyphal cell and hyphal cell, while *C. krusei* yeast cells can produce pseudohyphae but not true hyphae. On the other hand, there is no yeast-to-hyphae transition in *C. glabrata*^53^. Because the yeast form exists in all *Candida* species and is the form that initially colonizes host tissues and can circulate in the bloodstream throughout the body^17,54,55^, we focused on comparison of cell wall mannans extracted from the yeast form only. It is likely that mannans from various forms may have different effects, a possibility that would be worth exploring in future studies.

Our results show quantitative differences in DC responses to the cell wall mannans of these four distinct *Candida* species (Figs 1 and 2). This may be due to differences in their mannan structure and the composition of mannose residues^36,37,46–48^ (Figs. S8–12 and S17). *C. albicans* mannan consists of short-chain O-linked mannan oligosaccharides and branching N-linked mannan polysaccharide moieties. The N-linked mannan is composed of a long chain of α-1,6-linked mannose backbone connected with oligomannose side chains, mainly containing α-1,2-, α-1,3-, and β-1,2-linked mannose residues with a few phosphate groups^36,56^ (Fig. S8). *C. tropicalis* shares certain mannan moieties with *C. albicans* (Figs S8,9), and is antigenically identical to *C. albicans* serotype A^37,57,58^. Concurring with the similarity in mannan structure and composition of these two *Candida* species, we observed similar effects of *C. albicans* and *C. tropicalis* mannans on DCs (Figs 1 and 2), as well as similar T cell responses (Figs 6 and 7). In contrast, the cell wall mannan of *C. glabrata* contains small branches with low α-mannan content and one or two units of β-1,2-linked mannose residues^36,46^ (Fig. S10). Meanwhile, *C. krusei* mannan consists of a long chain of α-1,2-linked mannose backbone with one or two α-1,6-linked mannose residues located in the middle of the chain, and a small number of short side chains of α-1,2-linked mannose residues^48,50^ (Fig. S11). Presumably because the mannans of *C. glabrata* and *C. krusei* differ from those of *C. albicans* and *C. tropicalis*, they produce different effects on DC maturation and cytokine production (Figs. 1 and 2).

We found that *C. krusei* mannan induced DC maturation and massive cytokine production, which verified the DC state of high activation, and also led to increased apoptosis^34,35^. In contrast, *C. albicans* and *C. tropicalis* mannans had no effect on DC maturation, less effect on cytokine production, and did not induce DC apoptosis. *C. glabrata* mannan was a potent stimulus that induced DC maturation, but it failed to promote cytokine production or DC apoptosis (Fig. 3). Thus, high levels of DC activation and cytokine production may explain the increase in DC apoptosis in response to stimulation by *C. krusei* mannan. In addition, *C. albicans*, *C. tropicalis* and *C. glabrata* mannans, unlike *C. krusei* mannan, induced production of IFN-γ, which may act as an autocrine signal that enables the survival of BMDCs^59–61^.

Ample evidence supports the central role of CLRs and Syk in recognition of *Candida* mannans and protection against *C. albicans* infection^17,29^. Our results demonstrate that inhibition of Syk reduced the cell viability of unstimulated and β-glucan stimulated BMDCs; these results are consistent with those of previous studies^62–64^. Signal transduction via Syk is required for cell survival, as it leads to STAT3 phosphorylation, which mediates cell growth and differentiation^64^. Therefore, the mannans of *C. albicans*, *C. tropicalis* and *C. glabrata* may ligate to the receptors that activate Syk^17,19^, and this could partly explain the prevention of apoptosis in BMDCs (Fig. 3). In contrast, we found that *C. krusei* mannan mediated BMDC apoptosis via activation of MyD88 and TLR-2, which can transduce downstream signals through Fas-associated death domain protein (FADD) and caspase 8^65^. Of note, although DC apoptosis was increased in response to *C. krusei* mannan stimulation, the DCs retained their immunogenic functions that initiated T cell responses (Fig. 6).

*C. albicans* and *C. tropicalis* mannans induced high IFN-γ production in BMDCs (Fig. 2) and consequently skewed Th1 responses (Fig. 6). Both murine and human DCs are capable of producing IFN-γ upon the maturation^61,66^. IFN-γ is generally considered a key cytokine for Th1 induction since it epigenetically controls the expression and function of T-bet, a master transcription factor of Th1. IFN-γ also induces and maintains IL-12 receptor expression on T cells^67^. Thus, although a low level of IL-12 was detected in BMDCs stimulated with *C. albicans* and *C. tropicalis* mannans, the regulation of IL-12 receptor expression by IFN-γ probably helps Th1 polarization. In contrast to our finding, a previous study reported that ligation of *C. albicans* mannan to dectin-2 or mannose receptor promoted IL-6 and IL-23 production in APCs, and hence mediated Th17 response^20,68^. These contradicting results could be due to differences in the mannosyl composition of yeast cells grown under different conditions. The previous study cultured yeasts under specific conditions with limited carbon, low pH, and low temperature; the yeasts could not synthesize β-linked mannose^20^. In contrast, we grew the yeasts in an enriched media at 30 °C, which may have allowed them to synthesize the entire spectrum of α- and β-linked mannose components. It is likely that the presence of β-linked mannose in *C. albicans* mannan reduces inflammatory cytokine production in DCs^69^. In addition, the intracellular signal through DC-SIGN, which is a CLR that also recognizes *C. albicans* N-linked mannan^70^, appears to inhibit dectin-1-dependent Th17 generation, instead of favoring Th1 response, upon *Mycobacterium tuberculosis* infection^71^. Therefore, our observations support the hypothesis that cell wall mannan may play a role in *C. albicans* immune evasion by shifting the Th1/Th17 balance^72,73^.

TLR2 also serve as immune sensors that recognize specific mannose residues of *Candida* species^16^, and they are essential for host defense against *C. albicans* infection^31,32^. TLR2 directly engages with phospholipomannans^74^, which contain phospholipid and β-1,2-linked mannose residues. In addition, recognition of β-linked mannose by galectin-3 is associated with TLR2 activation^56^. The structure and composition of *C. krusei* mannan differ from these previously identified carbohydrate ligands of TLR2, but our results clearly show that *C. krusei* mannan was recognized by TLR2. The distinct structure of *C. krusei* mannan, which contains the interlaced α-1,2- and α-1,6-linked mannose residues^48,50^, possibly facilitates the specificity of the ligand-receptor binding.

Signal transduction via TLR2 and MyD88 in DCs has been shown to be involved in Th17 polarization^75,76^. Consistently, our findings demonstrate that activation of MyD88 in DCs by *C. krusei* mannan controlled Th1/Th17 switching by virtue of the polarizing cytokines IL-12 and IL-6 (Figs. 7 and 8). Although the roles of TLR2 and MyD88 in the *C. krusei* mannan-mediated Th17 response remain unclear, they may, at least in part, trigger IL-6, the key cytokine for Th17 generation^77,78^.

The *in vitro C. krusei* mannan-stimulated BMDCs exhibited the high production of both Th17 and Th1 polarizing cytokines, IL-6, IL-23, IFN-γ and IL-12 (Fig. 2), while the antigen-specific T cell responses to *C. krusei* mannan-activated DCs contributed toward the Th17 shift (Fig. 6). These results may be explained by the negative regulatory effect of IL-6 and IL-23 on Th1 differentiation. Previous report demonstrated that in the presence of IL-6 together with IFN-γ, CD4 T cells preferred Th17 differentiation. This response was resulted from the IL-6-mediated suppressor of cytokine signaling 1 (SOCS1) upregulation^79^, which consequently leaded to the interference in IFN-γ signaling in T cells. IL-23 also showed the suppressive effect on IFN-γ production from CD4 T cells, but via the inhibition of IL-12R signaling^80^. Therefore, it is possible that IL-6 and IL-23 interfere with IFN-γ and IL-12 signaling in T cells, and concomitantly regulate the switch from Th1 to Th17.

Our results imply that the cell wall mannan of *C. krusei* may help the pathogen to evade host defenses by augmenting DC apoptosis, but may also activate Th17 responses. Previous studies have shown that while Th17 plays a crucial role in protective immunity against *Candida* infections^25^, it can also cause severe pathology as it is a major driver of hyperinflammation and tissue damage^81–83^. Furthermore, alteration of the Th17 phenotype and function may be affected by changes in the microenvironment at various stages and progression of the fungal infection^81,84^. Thus, it is still uncertain whether the Th17 response to *C. krusei* mannan stimulation has a positive or negative effect on the host, and this warrants further investigation.

In conclusion, our study implies that the structure and composition of cell wall mannans from different *Candida* species crucially influence the regulation of host protective immunity and fungal immune evasion through differential activation of DCs. In this study, we selected a single strain of each species to examine immune responses related to inter-species mannan variation. However, *Candida* displays intra-species diversity in cell wall mannans, which may lead to the differential response of DCs, as well as adaptive immune responses. Therefore, DC responses to the mannans of other *Candida* strains, including clinical isolates, should be further investigated to reveal the host-*Candida* interaction in-depth. A thorough comprehension of *Candida* recognition by PRRs and subsequent induction of adaptive immunity may bring important contributions to the development of new diagnostic and therapeutic applications.

## Materials and Methods

### Animals

Six to eight-week-old female BALB/c and C57BL/6 mice were purchased from National Laboratory Animal Center, Mahidol University) and were housed at Chulalongkorn University Lab Animal Center. All animal experiments were performed in accordance with the protocol approved by the Institutional Animal Use and Care Committee of Chulalongkorn University Lab Animal Center (Protocol number 1573005). BALB/c mice were used for the overall experiments and C57BL/6 mice were used for OT-II experiments.

### Preparation of Candida cell wall mannan

*C. krusei* (ATCC 6258), *C. albicans* (ATCC 24433), *C. glabrata* (ATCC 2001) and *C. tropicalis* (ATCC 750) were selected as these strains are reference strains for quality control and antifungal drug susceptibility testing. All *Candida* were grown in YPD medium (Ajax Finechem, NSW, New Zealand) at 30 °C for 8 h with 200 rpm shaking. The fungal cultures were then diluted to OD_600_ of 0.05, and cultured at 30 °C for 14 h with 145 rpm shaking. With this culture condition, all *Candida* species grow as budding yeast-like cells^85–87^.

Mannan was extracted from *Candida* cell wall following the previously described method^88^. Briefly, *Candida* yeast cell pellets (100 g) were resuspended in 250 ml of citrate buffer (0.02 M, pH 7.0) and autoclaved at 121 °C for 90 min. The supernatant was collected by centrifugation, and the residual sediment was re-extracted with the same procedures. An equal volume of Fehling’s solution was added into the combined supernatants, and the mixture was stirred at 4 °C for overnight. The precipitate of mannan-copper complex was collected and dissolved in 6–8 ml of 3 N HCl, and the copper was then removed by washing in methanol-acetic acid (8:1, v/v) solution. After centrifugation, the mannan precipitate was collected and dissolved in sterile water. Subsequently, the mannan solution was dialyzed in sterile water for 48 h, and was further lyophilized for long-term storage. The amount of mannan was measured by phenol-sulfuric acid method^89^. All yeast culture and mannan preparation procedures were performed using endotoxin free water and containers. In addition, since the mannan extraction procedure required high temperature for a long period, nucleic acid and proteins are denatured and degraded. Therefore, there would be very less or no contamination of other PAMPs such as RNA and DNA.

### Generation and stimulation of BMDCs

BMDCs were generated following the previously described protocol^90^. Briefly, bone marrow cells were collected from femurs and tibias. The cells (1 × 10^6^ cells) were cultured in 24-well plates in 1 ml of RPMI 1640 (GIBCO, ThermoFisher Scientific, NY, USA) supplemented with 10% heat-inactivated fetal bovine serum (GIBCO), 0.2 mM Glutamax (GIBCO), 100 U/ml penicillin and 100 mg/ml streptomycin (HyClone, UT, USA), 10 ng/ml recombinant murine GM-CSF and 10 ng/ml recombinant murine IL-4 (Peprotech, NJ, USA). The cell culture were incubated under humidified atmosphere of 5% CO_2_ at 37 °C for 7 days. Half of culture media was replaced every 2 days.

On day 7, BMDCs were stimulated with mannan at the concentrations of 12.5, 25 and 50 μg/ml for 24–48 h. After stimulation, the culture supernatant was collected for cytokine measurement and the cells were harvested for flow cytometric analyses. Unstimulated BMDCs were used as the negative control, and BMDCs stimulated with 0.5 μg/ml of LPS (Sigma-Aldrich, MO, USA) were used as the positive control.

### Assessment of cell viability

BMDCs (1 × 10^5^ cells/well) were stimulated with *Candida* mannan in 96-well plates for 48 h. Next, the BMDCs were further incubated in the media containing 0.5 mg/ml of MTT (Life technologies, ThermoFisher Scientific, OR, USA) at 37 °C for 3 h. After removal of the culture media, the incorporated formazan crystals in viable cells were solubilized in dimethyl sulfoxide (DMSO; AMRESCO, OH, USA). The absorbance was measured using a microplate reader (Synergy H1, BioTek, VT, USA) at 570 nm. The percent of cell viability was calculated by normalization with the negative control.

### Treatment with inhibitors and TLR antagonists

For SYK and MYD88 inhibition, BMDCs were pre-incubated with 1 µM SYK inhibitor (R406; InvivoGen, CA, USA) for 30 min or 100 µM MYD88 inhibitor peptide (NBP2–29328; Novus Biologicals, CO, USA) for 24 h prior to stimulation. The negative control of SYK inhibitor and MYD88 inhibitor peptide was DMSO and control peptide, respectively.

For TLR blockade, BMDCs were pre-incubated with 20 µg/ml of anti-mouse/human TLR2 mAb (Biolegend, CA, USA) for 2 h prior to stimulation. Rat IgG was used as the isotype control.

### *In vivo* immunization and ex vivo re-stimulation assay

Mice were subcutaneously injection in the scruff of the neck with the mixture of *Candida* mannan (50 μg mannan per 1 g of body weight) and OVA (30 μg per animal; Sigma Aldrich) in 200 μl PBS at day 0 and day 7. On day 14, the cervical, axillary and brachial LNs were excised, digested with collagenase type IV (Sigma Aldrich) at 37°C for 30 min, and subsequently incubated with DNase I (Sigma Aldrich) at room temperature for 10 min. The cells were washed, and resuspended in RPMI 1640 supplemented with 10% heat-inactivated FBS, 0.2 mM Glutamax, 100 U/ml penicillin and 100 mg/ml streptomycin, and 55 μM 2ME (GIBCO). For the negative control, mice were immunized with OVA in PBS.

For *ex vivo* re-stimulation assay, LN cells (4 × 10^6^ cells) were cultured in 24-well plates in 1 ml media at the presence of 250 μg/ml OVA. The culture supernatant was collected at 48 h and 72 h after OVA stimulation.

### *In vitro* OT-II T cell stimulation

T cells from spleens of OT-II mice were enriched by immunomagnetic beads (Pan T Cell Isolation Kit II, mouse; Miltenyi Biotec, CA, USA). BMDCs were stimulated with *Candida* mannan for 12 h and pulsed with 500 μg/ml whole OVA protein overnight, and washed twice with the media to remove the mannan and the free OVA. Then, OT-II T cells were co-cultured with the OVA-pulsed BMDCs at T:DC ratio of 10:1. The supernatant was collected at 48 h and 72 h for cytokine determination.

In the MyD88 inhibitor and TLR-2 blockade experiment, BMDCs were pre-treated with control peptide, MyD88, control IgG or anti-mouse TLR2 mAbs as described above. The cells were then stimulated with 25 μg/ml of *C. albicans* or *C. krusei* mannan for 12 h, and pulsed with 500 μg/ml whole OVA protein overnight. Thereafter, all cells were washed twice with RPMI media to remove the inhibitor, *Candida* mannan and the free OVA. Then, OT-II T cells were co-cultured with the OVA-pulsed BMDCs.

### Flow cytometric analyses

All cells were incubated with Fc Block (anti-CD16/CD32; Biolegend) before labeling with the specific antibodies. To determine BMDC maturation, the cells were labeled with fluorochrome-conjugated mAb against murine CD11c, CD40, CD80, CD86 and I-A/I-E (Biolegend). For apoptosis assay, BMDCs were labeled with annexin V and 7-AAD (Biolegend). To assess immune cell population, LN cells were labeled with fluorochrome-conjugated mAb against murine B220, CD3ɛ, CD4, CD8a, CD44, CD62L, IFN-γ, IL-4 (Biolegend), IL-17A and FoxP3 (eBioscience, CA, USA). For intracellular staining, the cells were incubated with 50 ng/ml PMA (Sigma, MO, USA), 1 µM ionomycin (Sigma Aldrich), and 2 µg/ml Brefeldin A (PanReac AppliChem, Darmstadt, Germany) for 4 h prior to staining. The isotype matched antibodies were used as the control. All stained cells were acquired on a flow cytometry (CytoFLEX, Beckman Coulter, CA, USA) and the data were analyzed on Kaluza Flow Analysis Software (Beckman Coulter).

### Measurement of cytokines

Cytokines in the supernatant collected from BMDC, LN cell and OT-II T cell cultures were quantitated by standard sandwich ELISA using commercially available paired antibody sets for IL-1β, IL-4, IL-6, IL-10, IL-12, IL-17, IL-23, IFN-γ and TNF-α. The procedures were performed according to the manufacturer’s instructions (Biolegend and eBioscience).

### Statistical Analysis

All data values were expressed as mean ± SD, and the sample size was indicated in each figure legend. The statistical analysis was performed using one-way ANOVA with post-hoc Turkey HSD test for the comparison of 3–5 groups, and using Student’s T test for the comparison between 2 groups. Values of *p* < *0.05* were considered significant.

## Electronic supplementary material

Supplementary Information

## References

[1] Colombo AL, Junior JNA, Guinea J (2017). Emerging multidrug-resistant *Candida* species. Curr Opin Infect Dis.

[2] Kontoyiannis DP (2017). Antifungal Resistance: An Emerging Reality and A Global Challenge. J Infect Dis.

[3] Kim, S.H. *et al*. Risk factors and clinical outcomes of breakthrough yeast bloodstream infections in patients with hematological malignancies in the era of newer antifungal agents. *Med Mycol* (2017). 10.1093/mmy/myx038 PMC5896439 28525644

[4] Lortholary O (2017). The risk and clinical outcome of candidemia depending on underlying malignancy. Intensive Care Med.

[5] Tavernier E (2015). Development of echinocandin resistance in *Candida krusei* isolates following exposure to micafungin and caspofungin in a BM transplant unit. Bone Marrow Transplant.

[6] Vuichard D (2014). Weekly use of fluconazole as prophylaxis in haematological patients at risk for invasive candidiasis. BMC Infect Dis.

[7] Sharma U, Patel K, Shah V, Sinha S, Rathore VPS (2017). Isolation and Speciation of *Candid*a in Type II Diabetic Patients using CHROM Agar: A Microbial Study. J Clin Diagn Res.

[8] Mohammadi F, Javaheri MR, Nekoeian S, Dehghan P (2016). Identification of *Candida* species in the oral cavity of diabetic patients. Curr Med Mycol.

[9] Falahati M (2016). Characterization and identification of candiduria due to *Candida* species in diabetic patients. Curr Med Mycol.

[10] Pfaller MA (2008). *Candida krusei*, a multidrug-resistant opportunistic fungal pathogen: geographic and temporal trends from the ARTEMIS DISK Antifungal Surveillance Program, 2001 to 2005. J Clin Microbiol.

[11] Arendrup MC, Patterson TF (2017). Multidrug-Resistant *Candida*: Epidemiology, Molecular Mechanisms, and Treatment. J Infect Dis.

[12] Zhang L, Zhou S, Pan A, Li J, Liu B (2015). Surveillance of antifungal susceptibilities in clinical isolates of *Candida* species at 36 hospitals in China from 2009 to 2013. Int J Infect Dis.

[13] Forastiero A (2015). Rapid development of *Candida krusei* echinocandin resistance during caspofungin therapy. Antimicrob Agents Chemother.

[14] Kronen R, Lin C, Hsueh K, Powderly W, Spec A (2017). Risk Factors and Mortality Associated with *Candida krusei* Bloodstream Infections. Open Forum Infect Dis.

[15] Erwig LP, Gow NA (2016). Interactions of fungal pathogens with phagocytes. Nat Rev Microbiol.

[16] Brown GD (2011). Innate antifungal immunity: the key role of phagocytes. Annu Rev Immunol.

[17] Netea MG, Joosten LA, van der Meer JW, Kullberg BJ, van de Veerdonk FL (2015). Immune defence against *Candida* fungal infections. Nat Rev Immunol.

[18] Netea MG (2006). Immune sensing of *Candida albicans* requires cooperative recognition of mannans and glucans by lectin and Toll-like receptors. J Clin Invest.

[19] Robinson MJ (2009). Dectin-2 is a Syk-coupled pattern recognition receptor crucial for Th17 responses to fungal infection. J Exp Med.

[20] Saijo S (2010). Dectin-2 recognition of alpha-mannans and induction of Th17 cell differentiation is essential for host defense against *Candida albicans*. Immunity.

[21] Bain JM (2014). *Candida albicans* hypha formation and mannan masking of beta-glucan inhibit macrophage phagosome maturation. MBio.

[22] Nelson RD, Shibata N, Podzorski RP, Herron MJ (1991). *Candida* mannan: chemistry, suppression of cell-mediated immunity, and possible mechanisms of action. Clin Microbiol Rev.

[23] Chumpitazi BF (2014). Characteristic and clinical relevance of *Candida* mannan test in the diagnosis of probable invasive candidiasis. Med Mycol.

[24] Mikulska Małgorzata, Calandra Thierry, Sanguinetti Maurizio, Poulain Daniel, Viscoli Claudio (2010). The use of mannan antigen and anti-mannan antibodies in the diagnosis of invasive candidiasis: recommendations from the Third European Conference on Infections in Leukemia. Critical Care.

[25] Wuthrich M, Deepe GS, Klein B (2012). Adaptive immunity to fungi. Annu Rev Immunol.

[26] Zhu LL (2013). C-type lectin receptors Dectin-3 and Dectin-2 form a heterodimeric pattern-recognition receptor for host defense against fungal infection. Immunity.

[27] Kohatsu L, Hsu DK, Jegalian AG, Liu FT, Baum LG (2006). Galectin-3 induces death of *Candida* species expressing specific beta-1,2-linked mannans. J Immunol.

[28] Jouault T (2006). Specific recognition of *Candida albicans* by macrophages requires galectin-3 to discriminate Saccharomyces cerevisiae and needs association with TLR2 for signaling. J Immunol.

[29] Whitney PG (2014). Syk signaling in dendritic cells orchestrates innate resistance to systemic fungal infection. PLoS Pathog.

[30] Deng Z (2015). Tyrosine phosphatase SHP-2 mediates C-type lectin receptor-induced activation of the kinase Syk and anti-fungal TH17 responses. Nat Immunol.

[31] Netea MG (2002). The role of toll-like receptor (TLR) 2 and TLR4 in the host defense against disseminated candidiasis. J Infect Dis.

[32] Villamon E (2004). Toll-like receptor-2 is essential in murine defenses against *Candida albicans* infections. Microbes Infect.

[33] Arnold-Schrauf C, Berod L, Sparwasser T (2015). Dendritic cell specific targeting of MyD88 signalling pathways *in vivo*. Eur J Immunol.

[34] Takeuchi O, Akira S (2010). Pattern recognition receptors and inflammation. Cell.

[35] Kingeter LM, Lin X (2012). C-type lectin receptor-induced NF-kappaB activation in innate immune and inflammatory responses. Cell Mol Immunol.

[36] Shibata N, Kobayashi H, Suzuki S (2012). Immunochemistry of pathogenic yeast, *Candida* species, focusing on mannan. Proc Jpn Acad Ser B Phys Biol Sci.

[37] Kobayashi H (1994). Structures of cell wall mannans of pathogenic *Candida tropicalis* IFO 0199 and IFO 1647 yeast strains. Infect Immun.

[38] Rogers NC (2005). Syk-dependent cytokine induction by Dectin-1 reveals a novel pattern recognition pathway for C type lectins. Immunity.

[39] Jouault T (2003). *Candida albicans* phospholipomannan is sensed through toll-like receptors. J Infect Dis.

[40] Ibata-Ombetta S, Idziorek T, Trinel PA, Poulain D, Jouault T (2003). Role of phospholipomannan in *Candida albicans* escape from macrophages and induction of cell apoptosis through regulation of bad phosphorylation. Ann N Y Acad Sci.

[41] Kashem SW, Haniffa M, Kaplan DH (2017). Antigen-Presenting Cells in the Skin. Annu Rev Immunol.

[42] DuPage M, Bluestone JA (2016). Harnessing the plasticity of CD4^(+)^ T cells to treat immune-mediated disease. Nat Rev Immunol.

[43] Quintana FJ (2016). Old dog, new tricks: IL-6 cluster signaling promotes pathogenic TH17 cell differentiation. Nat Immunol.

[44] Revu S (2018). IL-23 and IL-1beta Drive Human Th17 Cell Differentiation and Metabolic Reprogramming in Absence of CD28 Costimulation. Cell Rep.

[45] Ozoren N (2006). Distinct roles of TLR2 and the adaptor ASC in IL-1beta/IL-18 secretion in response to *Listeria monocytogenes*. J Immunol.

[46] Takahashi S, Kudoh A, Okawa Y, Shibata N (2012). Significant differences in the cell-wall mannans from three *Candida glabrata* strains correlate with antifungal drug sensitivity. FEBS J.

[47] Jawhara S (2012). Murine model of dextran sulfate sodium-induced colitis reveals *Candida glabrata* virulence and contribution of beta-mannosyltransferases. J Biol Chem.

[48] Kogan G, Pavliak V, Sandula J, Masler L (1988). Novel structure of the cellular mannan of the pathogenic yeast *Candida krusei*. Carbohydr Res.

[49] Hirata T, Ishitani T (1976). Comparison of Proton Magnetic Resonance Spectra of Cell-wall Mannans of *Candida tropicalis* with Its Morphology. Agr Biol Chem.

[50] Nishikawa A, Shinoda T, Fukazawa Y (1982). Immunochemical determinant and serological specificity of *Candida krusei*. Mol Immunol.

[51] Shibata N, Suzuki A, Kobayashi H, Okawa Y (2007). Chemical structure of the cell-wall mannan of *Candida albicans* serotype A and its difference in yeast and hyphal forms. Biochem J.

[52] Lu Y, Su C, Liu H (2014). *Candida albicans* hyphal initiation and elongation. Trends Microbiol.

[53] Thompson DS, Carlisle PL, Kadosh D (2011). Coevolution of morphology and virulence in *Candida* species. Eukaryot Cell.

[54] Suzuki K, Kudo T, Hirai Y (2017). Phagocytized *Candida albicans* in the peripheral blood smear of a girl with Crohn disease. IDCases.

[55] Nadir E, Kaufshtein M (2005). Images in clinical medicine. *Candida albicans* in a peripheral-blood smear. N Engl J Med.

[56] Fradin C, Poulain D, Jouault T (2000). beta-1,2-linked oligomannosides from *Candida albicans* bind to a 32-kilodalton macrophage membrane protein homologous to the mammalian lectin galectin-3. Infect Immun.

[57] Hasenclever HF, Mitchell WO, Loewe J (1961). Antigenic studies of *Candida*. II. Antigenic relation of *Candida albicans* group A and group B to *Candida stellatoidea* and *Candida tropicalis*. Journal of bacteriology.

[58] Goins TL, Cutler JE (2000). Relative abundance of oligosaccharides in *Candida* species as determined by fluorophore-assisted carbohydrate electrophoresis. J Clin Microbiol.

[59] Frasca L (2008). IFN-gamma arms human dendritic cells to perform multiple effector functions. J Immunol.

[60] Zimmerman M (2010). IFN-gamma upregulates survivin and Ifi202 expression to induce survival and proliferation of tumor-specific T cells. PLoS One.

[61] Pan J (2004). Interferon-gamma is an autocrine mediator for dendritic cell maturation. Immunol Lett.

[62] Yousefi S, Hoessli DC, Blaser K, Mills GB, Simon HU (1996). Requirement of Lyn and Syk tyrosine kinases for the prevention of apoptosis by cytokines in human eosinophils. J Exp Med.

[63] Wilcox RA (2010). Inhibition of Syk protein tyrosine kinase induces apoptosis and blocks proliferation in T-cell non-Hodgkin’s lymphoma cell lines. Leukemia.

[64] Uckun FM, Qazi S, Ma H, Tuel-Ahlgren L, Ozer Z (2010). STAT3 is a substrate of SYK tyrosine kinase in B-lineage leukemia/lymphoma cells exposed to oxidative stress. Proc Natl Acad Sci USA.

[65] Aliprantis AO, Yang RB, Weiss DS, Godowski P, Zychlinsky A (2000). The apoptotic signaling pathway activated by Toll-like receptor-2. EMBO J.

[66] Yamaguchi N (2005). Interferon-gamma production by human cord blood monocyte-derived dendritic cells. Ann Hematol.

[67] Smeltz RB, Chen J, Ehrhardt R, Shevach EM (2002). Role of IFN-gamma in Th1 differentiation: IFN-gamma regulates IL-18R alpha expression by preventing the negative effects of IL-4 and by inducing/maintaining IL-12 receptor beta 2 expression. J Immunol.

[68] van de Veerdonk FL (2009). The macrophage mannose receptor induces IL-17 in response to *Candida albicans*. Cell Host Microbe.

[69] Ueno K (2013). The mannan of *Candida albicans* lacking beta-1,2-linked oligomannosides increases the production of inflammatory cytokines by dendritic cells. Med Mycol.

[70] Cambi A (2008). Dendritic cell interaction with *Candida albicans* critically depends on N-linked mannan. J Biol Chem.

[71] Zenaro E, Donini M, Dusi S (2009). Induction of Th1/Th17 immune response by *Mycobacterium tuberculosis*: role of dectin-1, Mannose Receptor, and DC-SIGN. J Leukoc Biol.

[72] Little CH (2000). Measurement of T-cell-derived antigen binding molecules and immunoglobulin G specific to *Candida albicans* mannan in sera of patients with recurrent vulvovaginal candidiasis. Infect Immun.

[73] Zhang SQ (2016). Mnn10 Maintains Pathogenicity in *Candida albicans* by Extending alpha-1,6-Mannose Backbone to Evade Host Dectin-1 Mediated Antifungal Immunity. PLoS Pathog.

[74] Li M, Chen Q, Shen Y, Liu W (2009). *Candida albicans* phospholipomannan triggers inflammatory responses of human keratinocytes through Toll-like receptor 2. Exp Dermatol.

[75] Aliahmadi E (2009). TLR2-activated human langerhans cells promote Th17 polarization via IL-1beta, TGF-beta and IL-23. Eur J Immunol.

[76] Liang J (2016). Inflammatory Th1 and Th17 in the Intestine Are Each Driven by Functionally Specialized Dendritic Cells with Distinct Requirements for MyD88. Cell Rep.

[77] Kashem SW (2015). *Candida albicans* morphology and dendritic cell subsets determine T helper cell differentiation. Immunity.

[78] Heink S (2017). Trans-presentation of IL-6 by dendritic cells is required for the priming of pathogenic TH17 cells. Nat Immunol.

[79] Diehl S (2000). Inhibition of Th1 differentiation by IL-6 is mediated by SOCS1. Immunity.

[80] Sieve AN, Meeks KD, Lee S, Berg RE (2010). A novel immunoregulatory function for IL-23: Inhibition of IL-12-dependent IFN-gamma production. Eur J Immunol.

[81] Whibley N, Gaffen SL (2014). Brothers in arms: Th17 and Treg responses in *Candida albicans* immunity. PLoS Pathog.

[82] Zelante T (2007). IL-23 and the Th17 pathway promote inflammation and impair antifungal immune resistance. Eur J Immunol.

[83] Krummey SM (2014). *Candida*-elicited murine Th17 cells express high CTLA-4 compared with Th1 cells and are resistant to costimulation blockade. J Immunol.

[84] Zelante T, De Luca A, D’Angelo C, Moretti S, Romani L (2009). IL-17/Th17 in anti-fungal immunity: what’s new?. Eur J Immunol.

[85] Kadosh D, Johnson AD (2005). Induction of the *Candida albicans* filamentous growth program by relief of transcriptional repression: a genome-wide analysis. Mol Biol Cell.

[86] Suzuki T (2006). Ethanol-induced pseudohyphal transition in the cells of *Candida tropicalis*: participation of phosphoinositide signal transduction. FEMS Yeast Res.

[87] Katiyar SK, Edlind TD (2001). Identification and expression of multidrug resistance-related ABC transporter genes in *Candida krusei*. Med Mycol.

[88] Kocourek J, Ballou CE (1969). Method for fingerprinting yeast cell wall mannans. Journal of bacteriology.

[89] Masuko T (2005). Carbohydrate analysis by a phenol-sulfuric acid method in microplate format. Analytical biochemistry.

[90] Inaba, K., Swiggard, W.J., Steinman, R.M., Romani, N. & Schuler, G. Isolation of dendritic cells. *Curr Protoc Immuno*l Chapter **3**, Unit3 7 (2001). 10.1002/0471142735.im0307s25 18432791

