# Supplementary material for: Cell wall mannan of Candida krusei mediates dendritic cell apoptosis and orchestrates Th17 polarization via TLR-2/MyD88-dependent pathway
Source: Sci Rep. 2018 Nov 20;8:17123. doi: 10.1038/s41598-018-35101-3 (PMC6244250; doi:10.1038/s41598-018-35101-3)
Supplement: Supplementary file 1 — Supplementary Information [file 41598_2018_35101_MOESM1_ESM.docx]

**Supplementary information**

**Cell wall mannan of *Candida krusei* mediates dendritic cell apoptosis and orchestrates Th17 polarization via TLR-2/MyD88-dependent pathway**

Thu Ngoc Yen Nguyen^1^, Panuwat Padungros^2^, Panachai Wongsrisupphakul^2^, Noppadol Sa-Ard-Iam^3^, Rangsini Mahanonda^3,4^, Oranart Matangkasombut^5,6^, Min-Kyung Choo^7^, Patcharee Ritprajak^6,8*^

^1^ Graduate program in Oral Biology, Faculty of Dentistry, Chulalongkorn University, Bangkok 10330, Thailand

^2^ Organic Synthesis Research Unit, Department of Chemistry, Faculty of Science, Chulalongkorn University, Phayathai Road, Patumwan, Bangkok 10330, Thailand

^3^ Immunology Laboratory, Faculty of Dentistry, Chulalongkorn University, Bangkok 10330, Thailand

^4^ Department of Periodontology, Faculty of Dentistry, Chulalongkorn University, Bangkok 10330, Thailand

^5^ Laboratory of Biotechnology, Chulabhorn Research Institute, Bangkok 10210, Thailand

^6^ Research Unit on Oral Microbiology and Immunology and Department of Microbiology, Faculty of Dentistry, Chulalongkorn University, Bangkok 10330, Thailand

^7^ Cutaneous Biology Research Center, Massachusetts General Hospital and Harvard Medical School, Charlestown, MA 02129

^8^ Oral Biology Research Center, Faculty of Dentistry, Chulalongkorn University, Bangkok, Thailand

^*^ Corresponding author

Patcharee Ritprajak, DDS, PhD, Assistant Professor

Department of Microbiology, Faculty of Dentistry, Chulalongkorn University

34 Henri-Dunant Road, Pathumwan, Bangkok, 10330, Thailand

Tel & Fax: +662-218-8680

E-mail address: Patcharee.R@chula.ac.th

**Supplementary methods**

**Acetylation, acetolysis and deacetylation of mannans**

The procedures were adapted from report previously described by method of Kocourek and Ballo^88^. Mannans (200 mg) were used in each chemical transformation. The deacetylated products were subjected to a Bio-Gel P-2 column (2.5 x 120 cm) purification and eluted with water at the rate of 0.56 mL/min at room temperature. The total carbohydrate content in each collected fraction was determined by the phenol-sulfuric acid method^89^.

**NMR spectroscopy**

NMR spectroscopy was carried out with a Bruker Avance 400 spectrometer at room temperature on mannan solution in D2O. 1H NMR spectra were recorded at 400 MHz with 128 scans. 13C NMR spectra were recorded at 100 MHz with 1024 scans^36-37,46,48-49^.

**Gel permeation chromatography**

Molecular weight of mannans were determined by using gel permeation chromatography (GPC). GPC was performed on Malvern Omnisec equipped with light scattering detector. Mannan 4 mg/mL 50 µL was subjected in each measurement and eluted with 10 mM PBS pH 7.4 at flow rate 1 mL/min. GPC column was spherical high purity 5 µm silica SEPAX column.


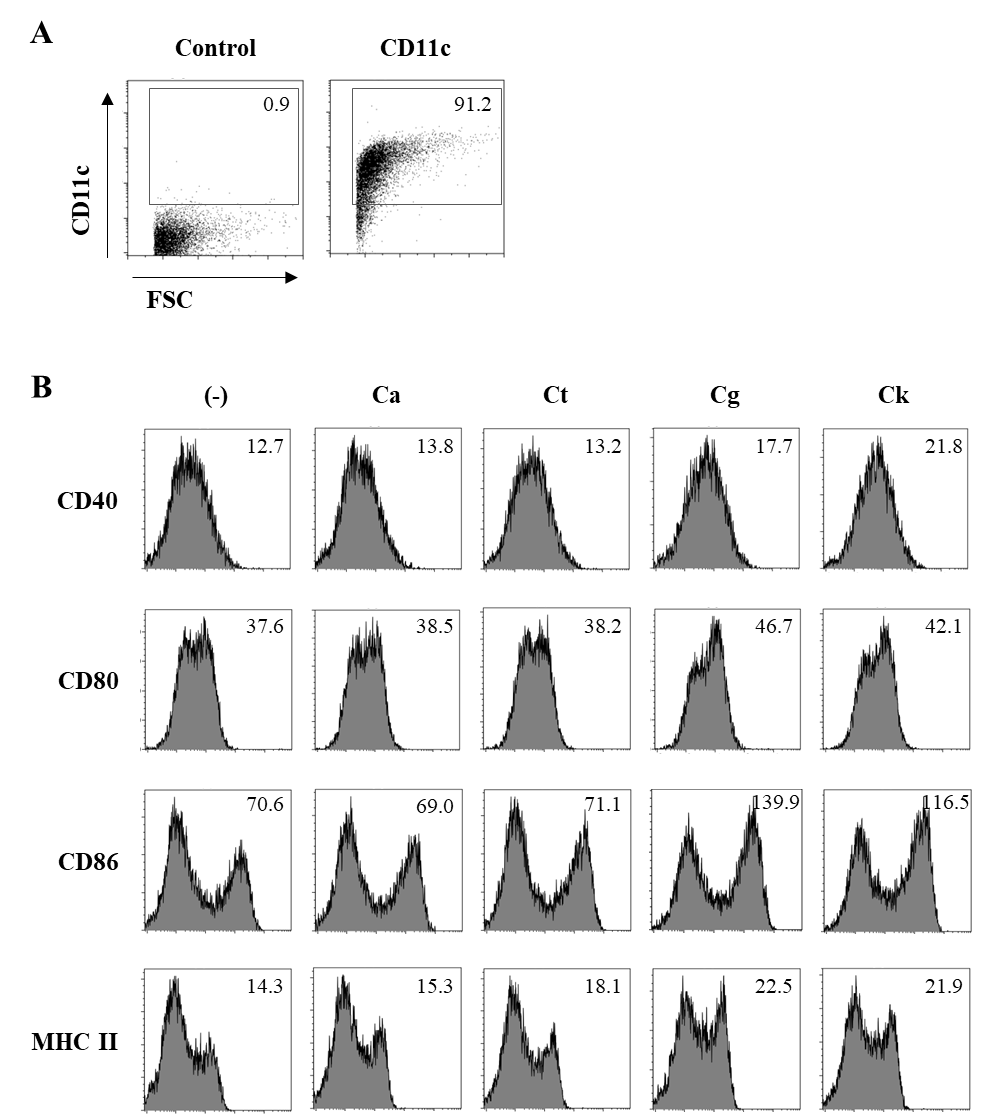


**Supplementary Figure 1** **Flow cytometric analysis of the expression level of DC maturation markers**

BMDCs were stimulated with *Candida* mannans, and the expression level of CD40, CD80, CD86 and MHC class II on CD11c^+^ cells was assessed using flow cytometry. The dot plot analysis of BMDC was first shown using side scatter (SSC) and forward scatter (FSC), and the live cells were gated. (a) DCs were identified by gating on CD11c^+^ population. The left panel is isotype control staining, and the right panel is CD11c staining. The number indicated the percentage of CD11c^+^ cells. (B) Histogram analysis of CD40, CD80, CD86 and MHC class II expression on CD11c^+^ cells. The numbers indicated the geometric MFI (-); unstimulated BMDCs, Ca, *C. albicans*; Ct, *C. tropicalis*; *Cg*, *C. glabrata*; Ck, *C. krusei*.


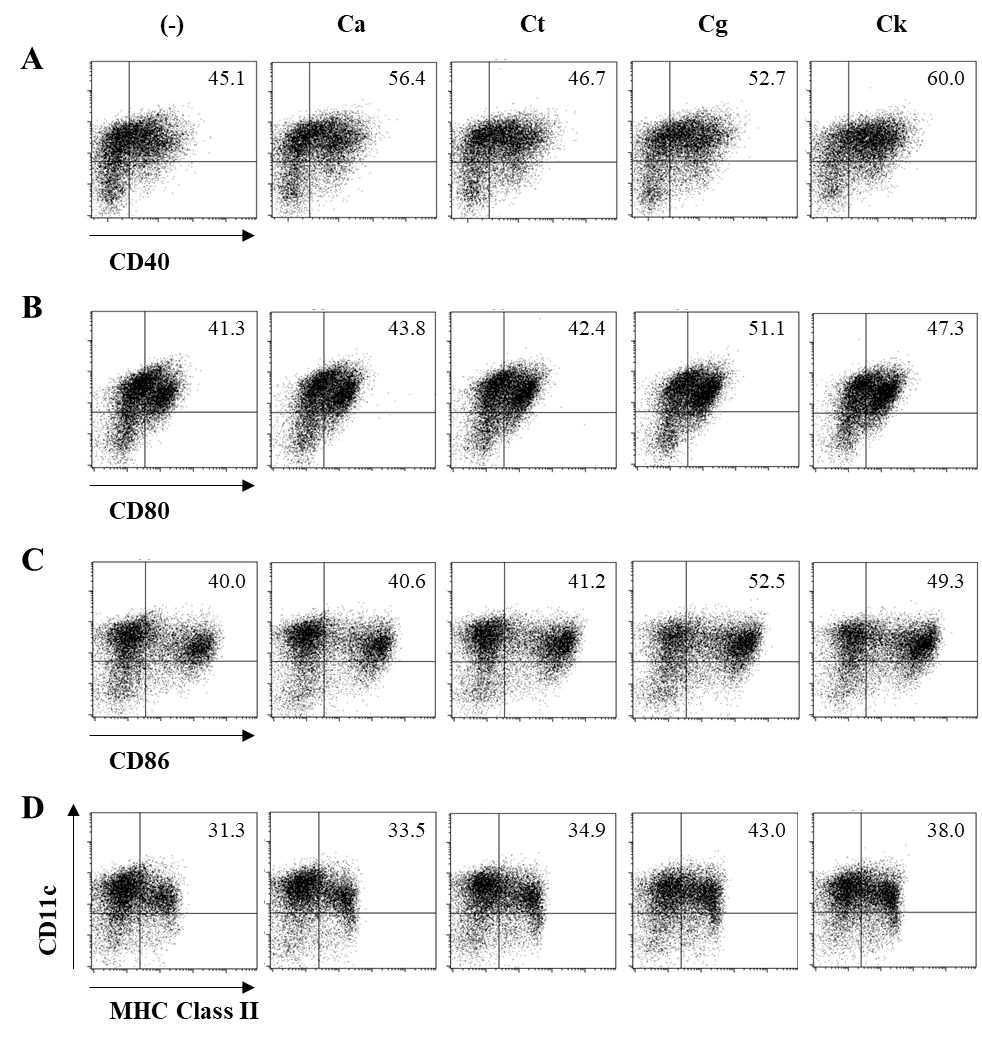


**Supplementary Figure 2 Flow cytometric analysis of the percentage of maturation marker expression on CD11c^+^ cells**

BMDCs were stimulated with *Candida* mannans, and the percentage of CD40^+^, CD80^+^, CD86^+^ and MHC class II^+^ cells within CD11c^+^ population were determined using flow cytometry. The live cells were gated based on SSC and FSC, and CD11c^+^CD40^+^, CD11c^+^CD80^+^, CD11c^+^CD86^+^ and CD11c^+^MHC class II^+^ cells were analyzed as shown in the dot plot. The numbers indicated the calculated percentage of double positive cells in CD11^+^ BMDCs. (-); unstimulated BMDCs, Ca, *C. albicans*; Ct, *C. tropicalis*; *Cg*, *C. glabrata*; Ck, *C. krusei*.

**
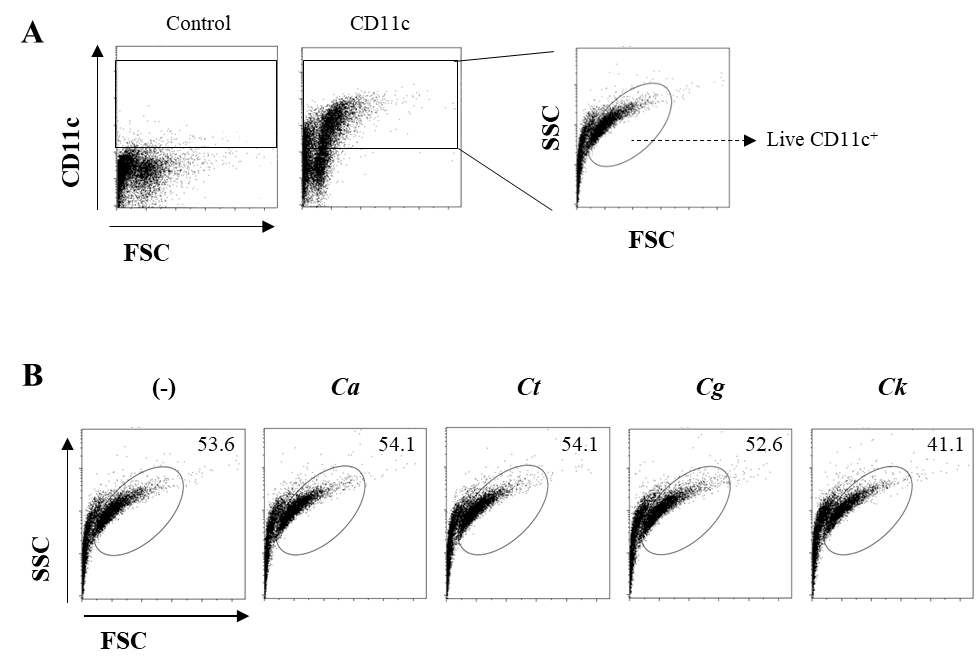
**

**Supplementary Figure 3** **Flow cytometric analysis of live CD11c^+^ DCs**

BMDCs were stimulated with *Candida* mannans, and then were stained with CD11c (A) CD11c^+^ population was gated and the live cells were identified based on SSC and FSC. (B) Showed the dot plot analysis of live DCs when stimulated with *Candida* mannans. The numbers indicated the percentage of live CD11c^+^ cells. (-); unstimulated BMDCs, Ca, *C. albicans*; Ct, *C. tropicalis*; *Cg*, *C. glabrata*; Ck, *C. krusei*.


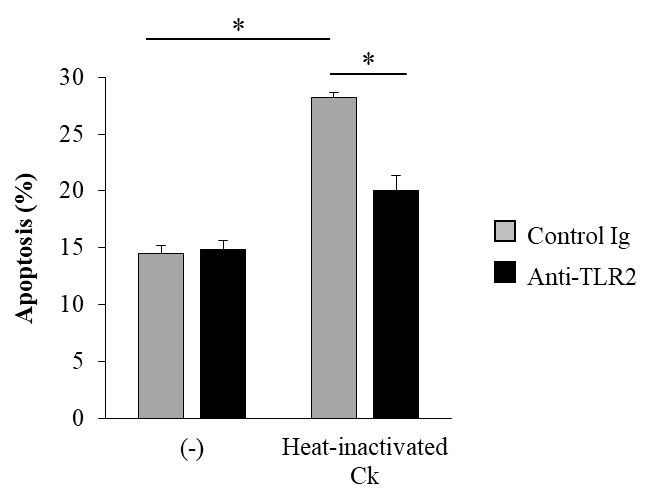


**Supplementary Figure 4** **Blockade of TLR-2 in BMDCs stimulated with heat-inactivated *C. krusei***

BMDCs were pre-treated with control IgG or anti-mouse TLR2 mAbs, and the cells were incubated with heat-inactivated *C. krusei* at the DC/yeast ratio of 1:4 for 24 h. Subsequently, the cells were stained with CD11c, Annexin V and 7AAD. Annexin V^+^, and Annexin V^+^7AAD^+^ were identified as apoptotic fraction. n = 4. * *p*<0.05. (-), unstimulated BMDCs; Ck, *C. krusei*.

**
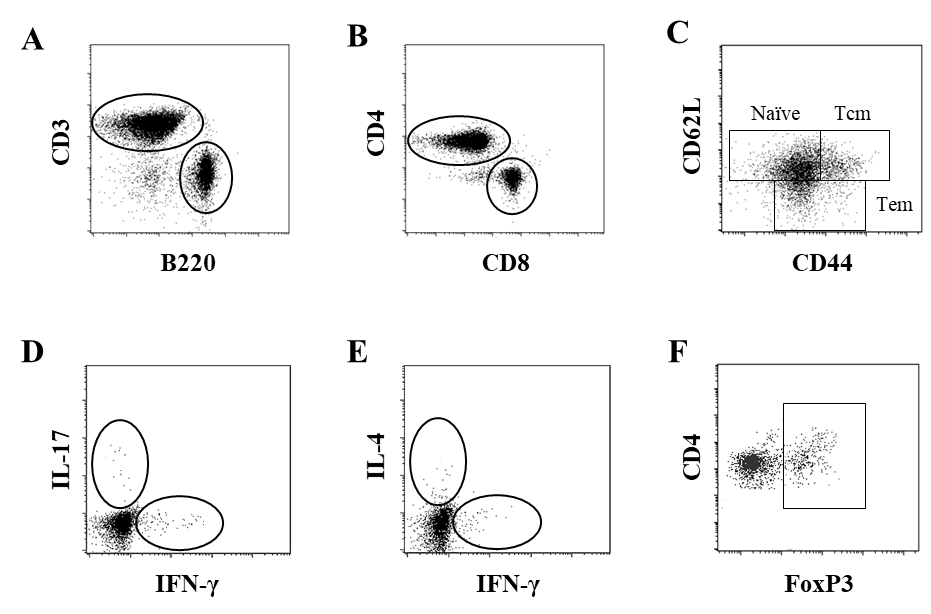
**

**Supplementary Figure 5** **Flow cytometric analysis of immune cell population**

Mice were subcutaneously immunized with the mixture of OVA and *Candida* mannans at day 0 and day 7. At day 14, the immune cell population in RLN cells were investigated using flow cytometric analysis. (A) Show the dot plot analysis of CD3^+^ and B220^+^ cells. (B) CD3^+^ cells were gated, and CD4^+^ and CD8^+^ cells were subsequently identified. To determine the naïve and memory phenotype, CD3^+^CD4^+^ cells were first gated, and (C) CD62L^+^CD44^lo^ (naïve), CD62L^-^CD44^+^ (effector memory T cells or Tem), and CD62L^+^CD44^hi^ (central memory T cells or Tcm) were then identified. To determine T helper cell subpopulation, CD3^+^CD4^+^ cells were gated, and (D) IL-17^+^ and IFN-γ^+^ or (E) IL-4^+^ and IFN-γ^+^ or (F) CD4^+^FoxP3^+^ T cells were then identified.

**
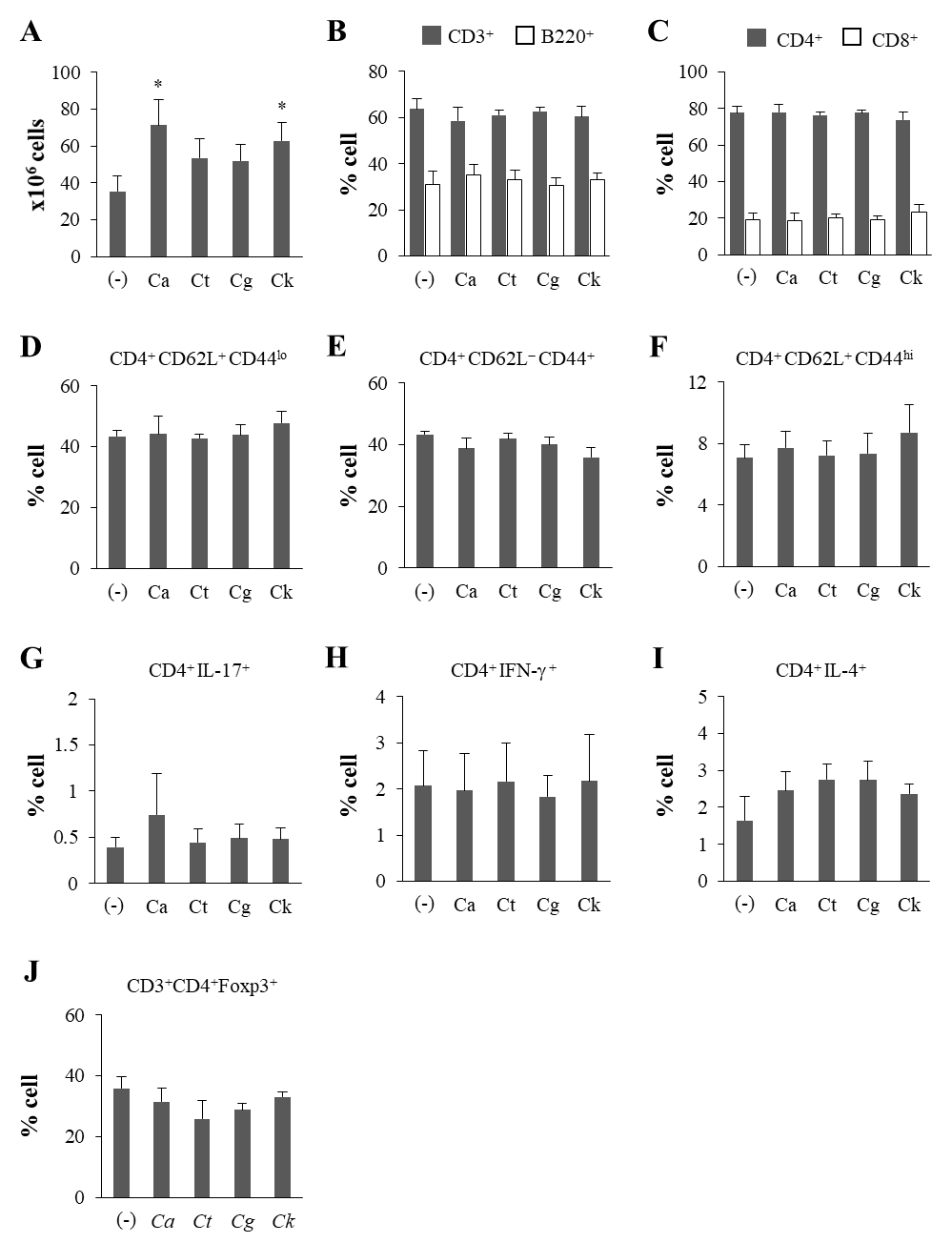
**

**Supplementary Figure 6** **Comparison of cell number and immune cell population in the RLNs in response to OVA and *Candida* mannan immunization**

Mice were subcutaneously immunized with the mixture of OVA and *Candida* mannans at day 0 and day 7. At day 14, the immune cell population in RLN cells were investigated using flow cytometric analysis as described in Fig S4. (A) Number of total LN cells. (B) to (J) Show the percentage of (B) T cells (CD3^+^) and B cells (B220^+^), (C) CD4^+^ and CD8^+^ within CD3^+^ T cells, (D) naïve CD4^+^ T cells, (E) CD3^+^CD4^+^ Tem, (F) CD3^+^CD4^+^ Tcm , (G) CD4^+^ IL-17^+^ T cells (H) CD4^+^ IFN-γ^+^ T cells (I) CD4^+^ IL-4^+^ T cells (J) CD3^+^CD4^+^FoxP3^+^ T cells.

**
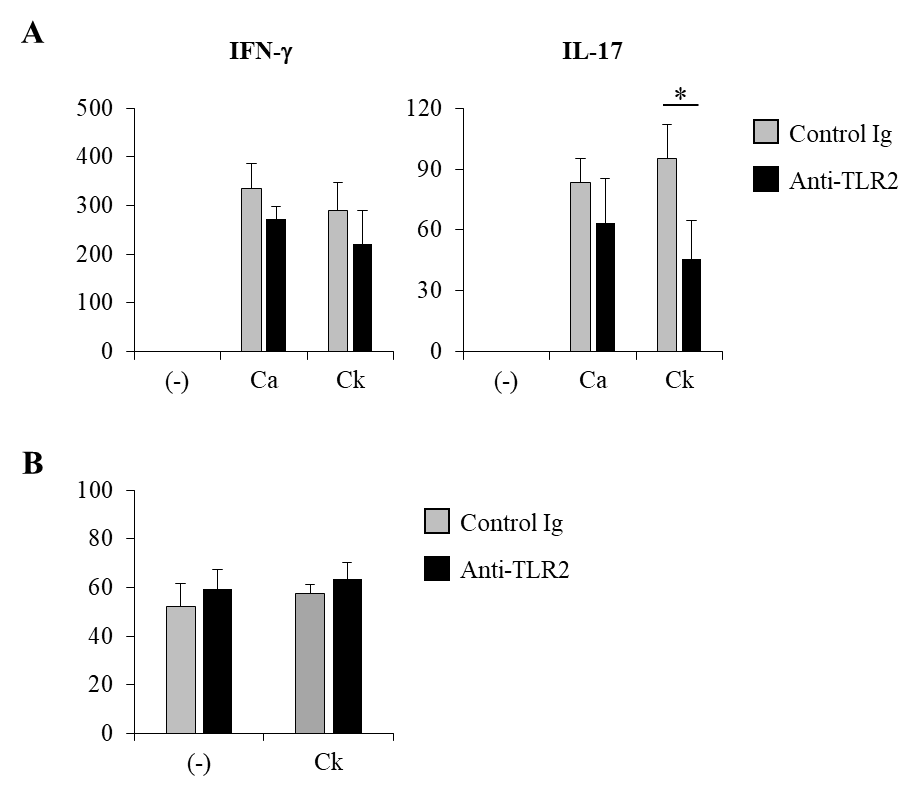
**

**Supplementary Figure 7 Blockade of TLR-2 in *C. krusei* mannan-stimulated DCs**

(A) BMDCs were pre-treated with control IgG or anti-mouse TLR2 mAbs, and the cells were stimulated with 25 μg/ml of *C. albicans* or *C. krusei* mannan. Subsequently, unstimulated and mannan-stimulated BMDCs were pulsed with OVA, and the BMDCs were co-cultured with OT-II T cells for 48 h. The levels of IFN-γ and IL-17 in the culture supernatant were determined by ELISA. (-), T cell incubated with OVA as a negative control. (B) BMDCs were pre-treated with control IgG or anti-mouse TLR2 mAbs. Then, the cells were stimulated with 25 μg/ml of *C. krusei* mannan, and the levels of IL-1β in the culture supernatant were determined by ELISA. (-), unstimulated BMDCs. n = 3. * *p*<0.05. Ca, *C. albicans*; Ck, *C. krusei*.

^13^C NMR spectrum (100 MHz, D_2_O) of *C. albicans* mannan

^1^H NMR spectrum (400 MHz, D_2_O) of *C. albicans* mannan

**Supplementary Figure 8** **NMR analysis of *C. albicans* mannan**

^13^C NMR signals at 103.31, 101.91, and 99.25 ppm corresponds to anomeric carbons (C1) of α-1,3-linked, α-1,2-linked, and α-1,6-linked mannose, respectively.

^13^C NMR spectrum (100 MHz, D_2_O) of *C. tropicalis* mannan

^1^H NMR spectrum (400 MHz, D_2_O) of *C. tropicalis* mannan

**Supplementary Figure 9** **NMR analysis of *C. tropicalis* mannan**

^13^C NMR signals at 100.90, 100.54, and 99.22 ppm corresponds to anomeric carbons (C1) of α-1,3-linked, α-1,2-linked, and α-1,6-linked mannose, respectively.

^13^C NMR spectrum (100 MHz, D_2_O) of *C. glabrata* mannan

^1^H NMR spectrum (400 MHz, D_2_O) of *C. glabrata* mannan

**Supplementary Figure 10** **NMR analysis of *C. glabrata* mannan**

^1^H NMR signals at 5.57, 5.55, 5.30, 5.15, 5.10, 5.06 ppm corresponds to anomeric proton (H1) with α-linked and signal at 4.85 ppm belongs to anomeric proton (H1) with β -linked mannose.

**A**

^13^C NMR spectrum (400 MHz, D_2_O) of *C. krusei* mannan

^1^H NMR spectrum (100 MHz, D_2_O) of *C. krusei* mannan

**B**

^1^H NMR spectrum (400 MHz, D_2_O) of deacetylated manno-oligosaccharide from

*C. krusei* mannan after Bio-Gel P-2 purification

**Supplementary Figure 11** **NMR analysis of *C. krusei* mannan**

(A) ^13^C NMR signals at 103.31, 101.76, and 99.28 ppm corresponds to anomeric carbons (C1) of terminal non-reducing, 2-substituted, and 2,6-disubstituted mannose, respectively. (B) ^1^H NMR of the manno-oligosaccharide contains signals at 5.36, 5.27, and 5.01 ppm for anomeric proton (H1) of non-reducing end, α-1,2-linked, and reducing end, respectively.

**
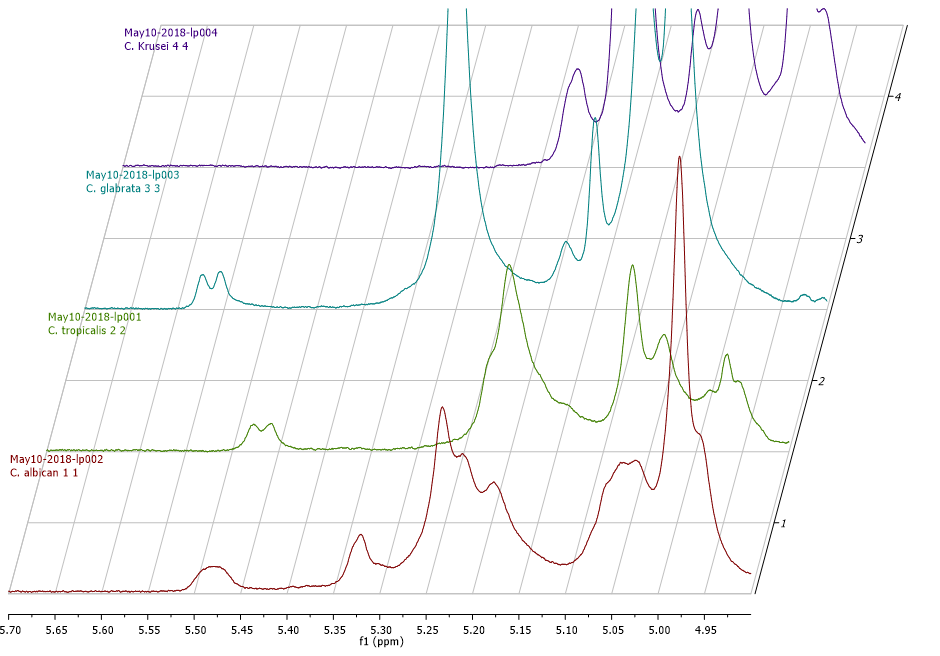
**

**Supplementary Figure 12 ^1^H NMR comparison of *C. albicans, C. tropicalis, C. glabrata, and C. krusei* mannan between 5.70–4.90 ppm**


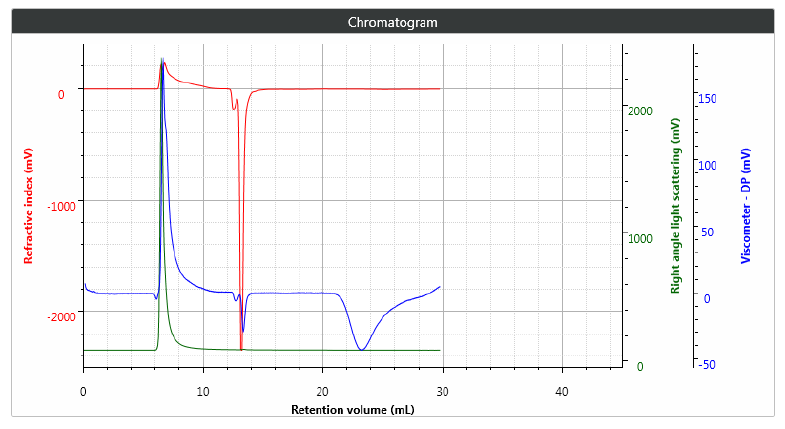

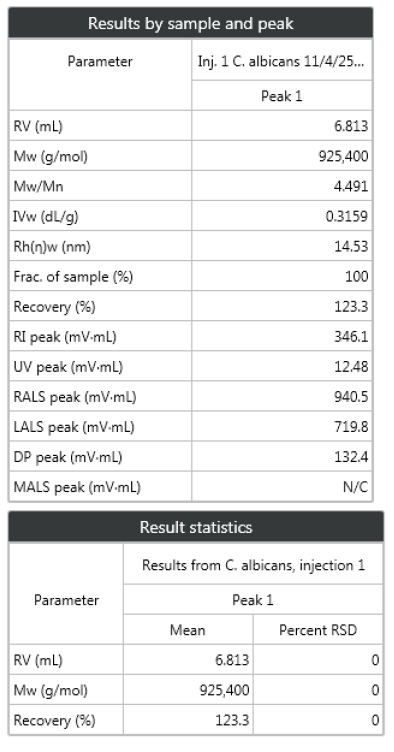


**Supplementary Figure 13 Gel permeation chromatography (GPC) analysis of *C. albicans* mannan**

Molecular weight of *C. albicans* mannan was 925,400 g/mol.


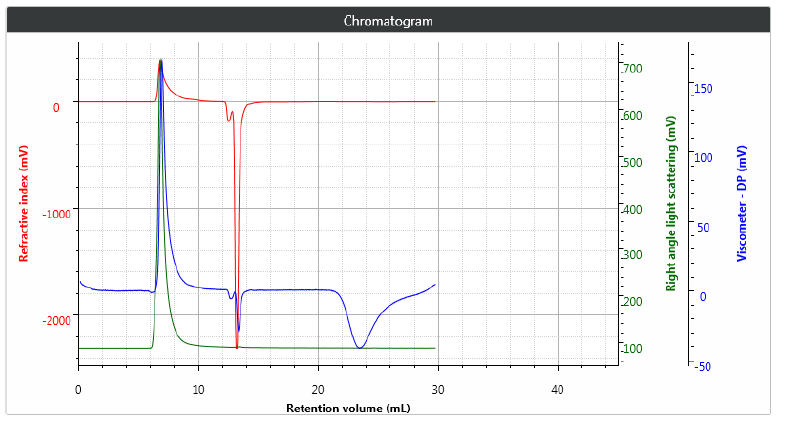

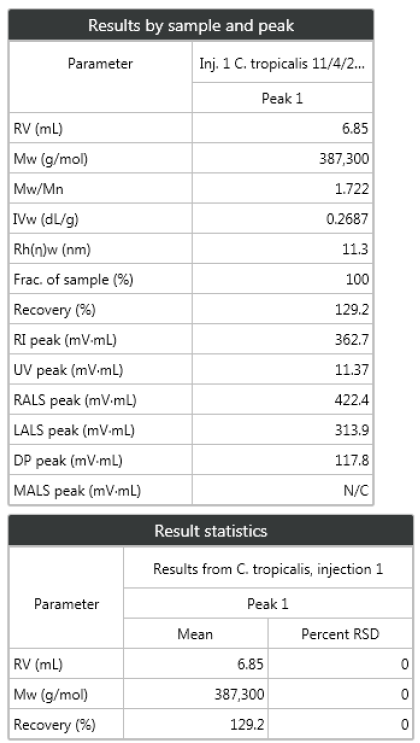


**Supplementary Figure 14 Gel permeation chromatography (GPC) analysis of *C. tropicalis* mannan**

Molecular weight of *C. tropicalis* mannan was 387,300 g/mol.


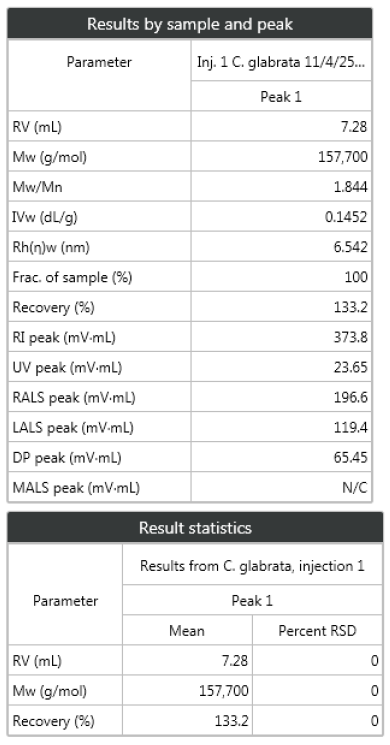

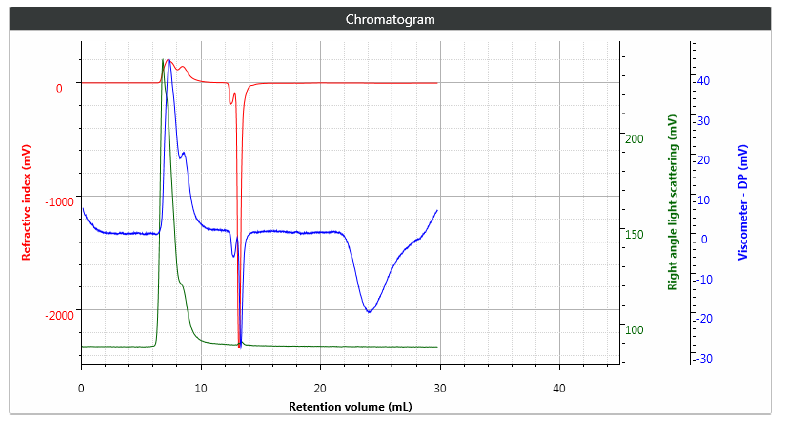


**Supplementary Figure 15 Gel permeation chromatography (GPC) analysis of *C. glabrata* mannan**

Molecular weight of *C. glabrata* mannan was 157,700 g/mol.


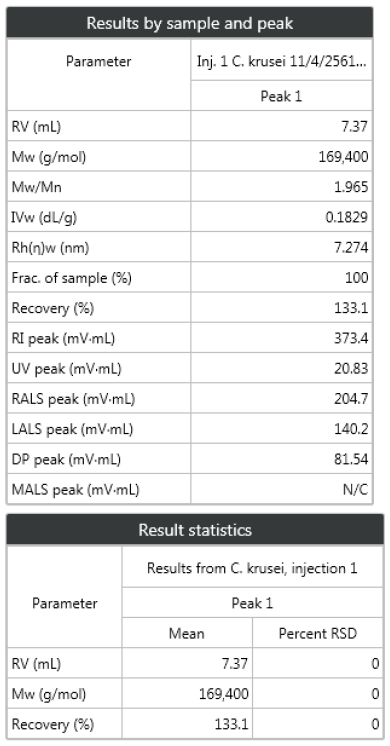

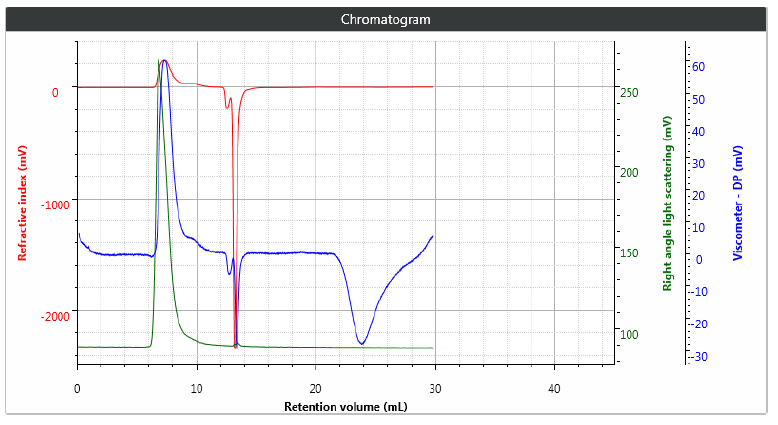


**Supplementary Figure 16 Gel permeation chromatography (GPC) analysis of *C. krusei* mannan**

Molecular weight of *C. krusie* mannan was 169,400 g/mol.

**
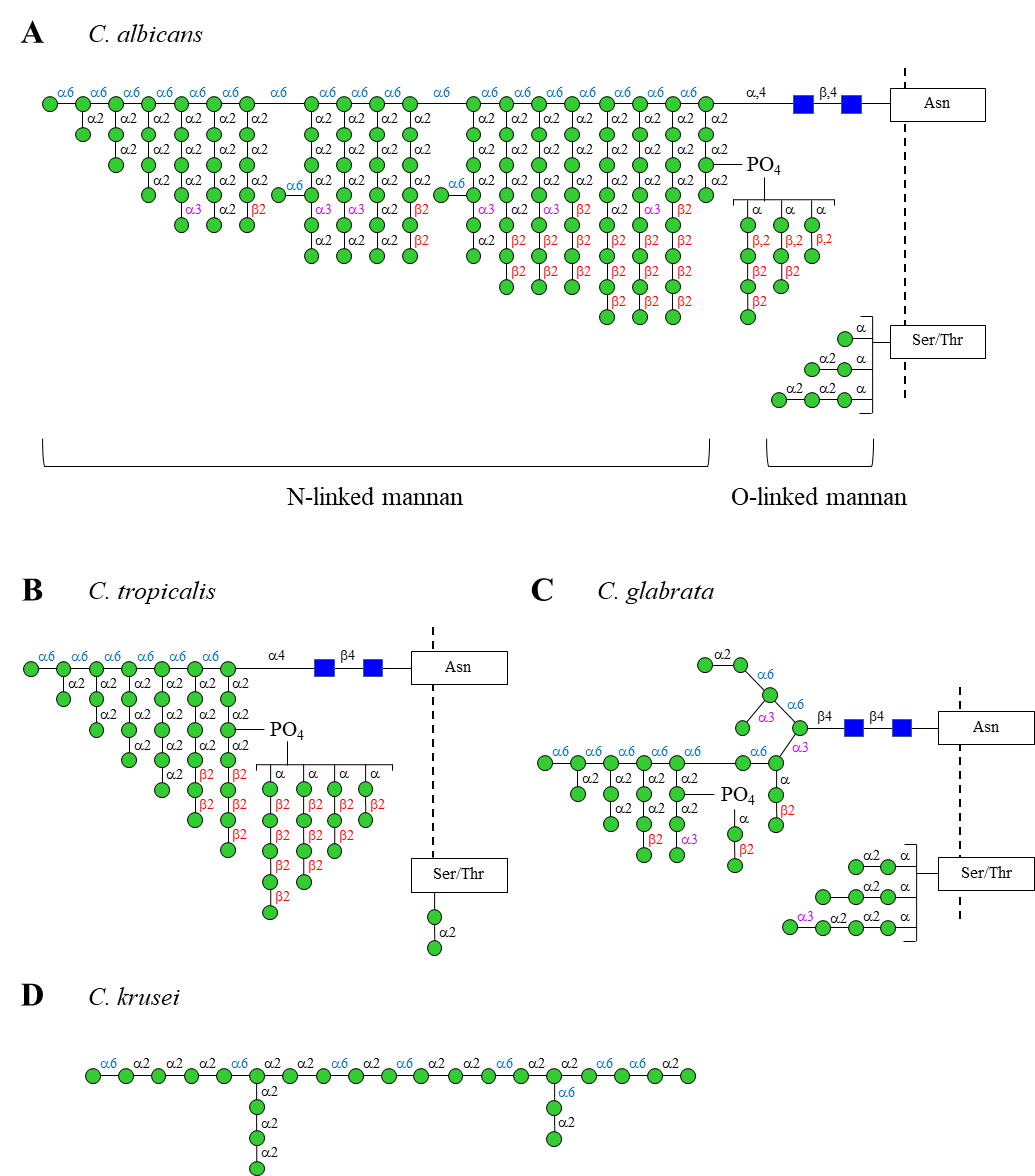
**

**Supplementary Figure 17** **The possible structure of cell wall mannan of *Candida* species**

The illustration showed the predicted structure of cell wall mannan of the yeast form of (A) *C. albicans*^36^ (B) *C. tropicalis*^37^ (C) *C. glabrata*^48,49^ and (D) *C. krusei*^50^.
